# Catestatin improves insulin sensitivity by attenuating endoplasmic reticulum stress: *In vivo* and *in silico* validation

**DOI:** 10.1016/j.csbj.2020.02.005

**Published:** 2020-02-22

**Authors:** Abhijit Dasgupta, Gautam K. Bandyopadhyay, Indrani Ray, Keya Bandyopadhyay, Nirmalya Chowdhury, Rajat K. De, Sushil K. Mahata

**Affiliations:** a VA San Diego Healthcare System, 3350 La Jolla Village Drive, San Diego, CA 92161, USA; b Machine Intelligence Unit, Indian Statistical Institute, 203 B.T. Road, Kolkata 700108, India; c Department of Medicine, University of California, San Diego, 9500 Gilman Drive, La Jolla, CA 92093-0732, USA; d Department of Computer Science & Engineering, Jadavpur University, Kolkata 700032, India; e Department of Data Science, School of Interdisciplinary Studies, University of Kalyani, Kalyani, Nadia 741235, West Bengal, India

**Keywords:** Chromogranin A, Catestatin, Endoplasmic reticulum stress, Insulin sensitivity, Obesity, PID controller

## Abstract

Obesity is characterized by a state of chronic, unresolved inflammation in insulin-targeted tissues. Obesity-induced inflammation causes accumulation of proinflammatory macrophages in adipose tissue and liver. Proinflammatory cytokines released from tissue macrophages inhibits insulin sensitivity. Obesity also leads to inflammation-induced endoplasmic reticulum (ER) stress and insulin resistance. In this scenario, based on the data (specifically patterns) generated by our *in vivo* experiments on both diet-induced obese (DIO) and normal chow diet (NCD) mice, we developed an *in silico* state space model to integrate ER stress and insulin signaling pathways. Computational results successfully followed the experimental results for both DIO and NCD conditions. Chromogranin A (CgA) peptide catestatin (CST: ${\text{hCgA}}_{352-372}$) improves obesity-induced hepatic insulin resistance by reducing inflammation and inhibiting proinflammatory macrophage infiltration. We reasoned that the anti-inflammatory effects of CST would alleviate ER stress. CST decreased obesity-induced ER dilation in hepatocytes and macrophages. On application of Proportional-Integral-Derivative (PID) controllers on the *in silico* model, we checked whether the reduction of phosphorylated PERK resulting in attenuation of ER stress, resembling CST effect, could enhance insulin sensitivity. The simulation results clearly pointed out that CST not only decreased ER stress but also enhanced insulin sensitivity in mammalian cells. *In vivo* experiment validated the simulation results by depicting that CST caused decrease in phosphorylation of UPR signaling molecules and increased phosphorylation of insulin signaling molecules. Besides simulation results predicted that enhancement of AKT phosphorylation helps in both overcoming ER stress and achieving insulin sensitivity. These effects of CST were verified in hepatocyte culture model.

## Highlights

• An endogenous peptide catestatin alleviates obesity-induced ER stress.
• Alleviation of ER stress by catestatin improves insulin sensitivity.
• PID controller based model of ER stress is supported by experimental findings.
• It predicts AKT phosphorylation achieves insulin sensitivity overcoming ER stress.

## Graphical abstract

**Figure:**
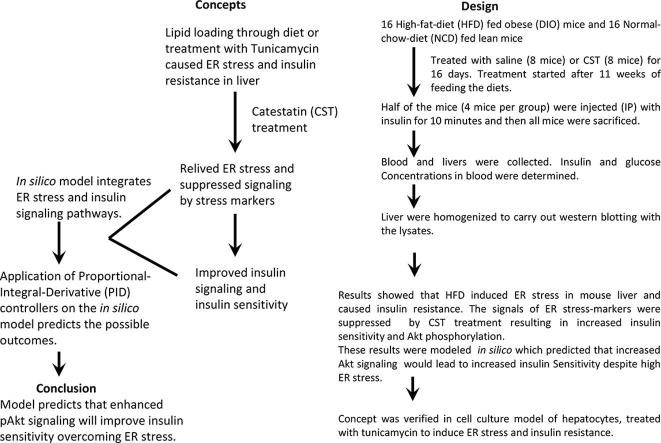

## 1 Introduction

The liver maintains whole body homeostasis by regulating critical metabolic, secretory and excretory functions. Calcium storage, protein and lipid synthesis along with protein folding are the key functions of the endoplasmic reticulum (ER) [1]. Hepatocytes (representing up to 70% of entire liver cells) contain both rough and smooth ER, which perform the myriad of metabolic functions [2]. The smooth ER synthesizes not only the majority of the membrane lipids but also their intermediates such as cholesterol, ceramides, and glycerophospholipids [3], [4]. Both rodents [5], [6] and humans [7], [8], [9] accumulate ceramides in tissues and plasma [10], which inhibits insulin action by decreasing phosphorylation of AKT and consequent inhibition of glucose uptake. Ceramide also activates nuclear factor-κ-B (NF-κB)-tumor necrosis factor-α (TNF-α) axis and induces inflammation [5], [6]. The rough ER controls the synthesis and maturation of proteins, which comprises up to 40% of cells proteome of the secretory pathway [11]. Ribosomes perform the translation of proteins on the cytosolic surface of the ER [12], and sec61 complex translocates the unfolded polypeptide into the ER lumen [13] where they undergo N-glycosylation and folding into secondary or tertiary structures. The rough ER lumen is enriched with high concentrations of calcium, molecular chaperones and folding enzymes, which facilitates protein folding and maturation [14]. Non-native proteins are recognized by the ER associated degradation (ERAD) quality control system and are degraded by the cytosolic ubiquitin-proteasome system [15], [16]. ER stress is characterized by the accumulation of misfolded or unfolded proteins in response to environmental insults, increased protein synthesis and reduced secretory efficacy [17], [18]. Homeostasis is restored by the ER stress-induced activation of the adaptive unfolded protein response (UPR). The following three ER localized proteins initiate UPR signaling in mammalian cells: double-stranded RNA-dependent protein kinase-like ER kinase (PERK) - eukaryotic translation initiation factor 2α (eIF2α), inositol-requiring 1α (IRE1α) - X-box-binding protein (XBP1), and activating transcription factor-6α (ATF6α) [19]. When physiological UPR becomes chronically activated, ER stress occurs. Thus, the chronic activation of the UPR has been reported in human obesity and non-alcoholic fatty liver disease (NAFLD), and in the adipose and/or liver tissue of dietary and genetic murine models of obesity [20], [21], [22], [23], [24], [25], [26].

The levels of free fatty acids (FFA), insulin, glucose, proinflammatory cytokines and ceramides are increased in blood of obese rodents and humans, which activates the innate immune system resulting in a chronic low-grade inflammation of white adipose tissue [10], [27] and the subsequent development of insulin resistance on other peripheral tissues, including the skeletal muscle, adipose and liver [28], [29], [30]. Thus, obesity aggravates both inflammation and ER stress.

We develop an *in silico* state space model corresponding to the integrated ER stress and insulin signaling pathways. Subsequently, we simulate the model by applying external inputs responsible for both high ER stress (diet-induced obese (DIO) condition) and normal condition (normal chow diet (NCD) or control). Here we find that the model follows the experimental cellular behavior of both DIO and NCD mice.

**Fig. 1:**
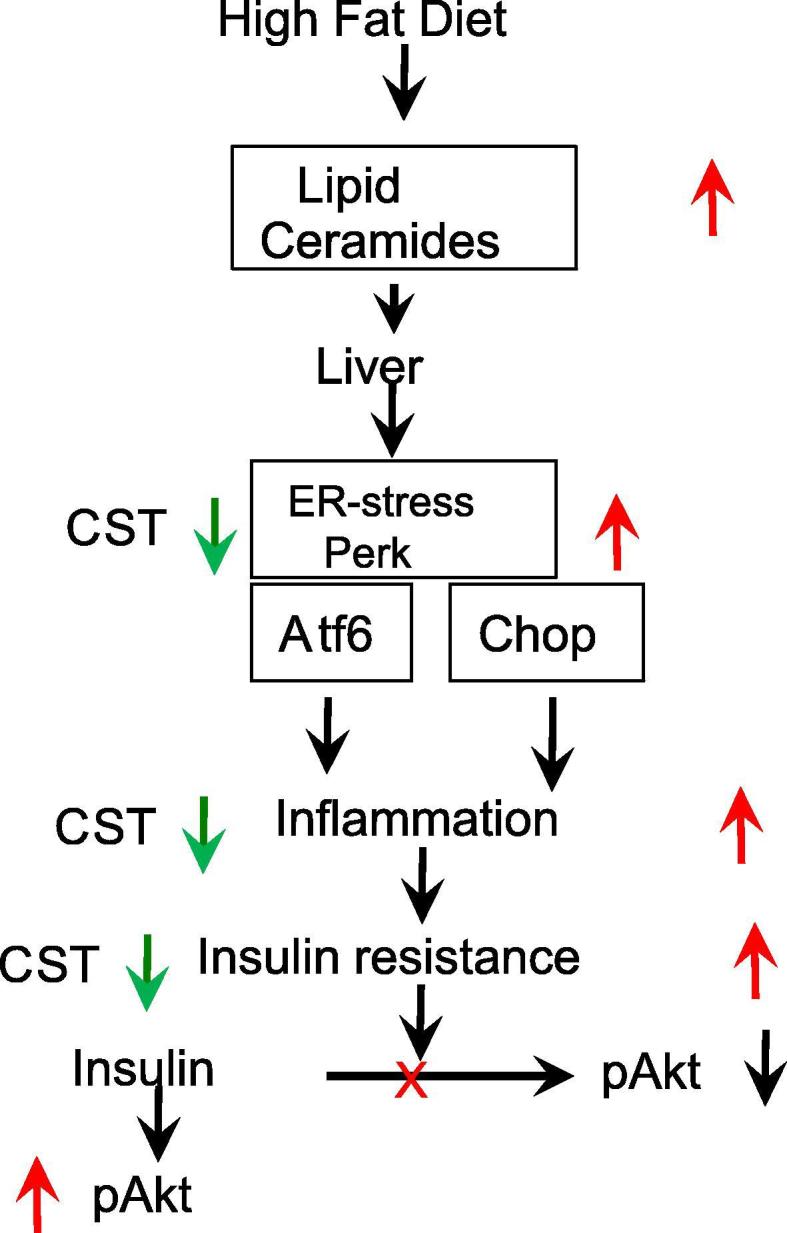
CST improves insulin sensitivity by suppressing ER stress. Black arrows indicate flow of the pathway, Red arrows indicate increase and Green arrows indicate decrease. In silico modelling will integrate these signaling features and predict an outcome. Here, ‘X’ indicates inhibition. (For interpretation of the references to colour in this figure legend, the reader is referred to the web version of this article.)

A recent investigation has shown that the chromogranin A (CgA) peptide catestatin (CST: human ${\mathrm{CgA}}_{352-372}$) [31], [32] improves hepatic insulin sensitivity in DIO mice as well as in insulin-resistant CST knockout mice by reducing inflammation and inhibiting infiltration of macrophages [33]. Since ER stress activates the inflammatory response [34], [35], [36] and the inflammatory response in turn also activates ER stress [37], we reasoned that one additional mechanism by which CST can improve insulin resistance in DIO mice is by alleviating ER stress (Fig. 1).

We have tested the above hypothesis by applying two Proportional-Integral-Derivative (PID) controllers on the *in silico* state space model to control the signal level of probable drug targets/markers, such as tyrosine phosphorylated insulin receptor (IRpY), tyrosine phosphorylated insulin receptor substrate (IRSpY), and phosphorylated Protein Kinase B (pAKT) along with phosphorylated PERK (pPERK), to low/high values. The simulation results clearly indicated that reduction of pPERK resulting in attenuation of ER stress, which was achieved by applying CST as shown by the experimental results, led to high insulin sensitivity. Besides, IRpY and IRSpY could not be the significant markers to be targeted for both reduced ER stress and higher insulin sensitivity. Thus, computational results demonstrate that the reduction of ER stress on application of CST is one of the potential factors to enhance insulin sensitivity in mammalian cells. This computational hypothesis is confirmed by *in vivo* experiments. Here phosphorylation of JNK significantly decreases due to chronic treatment with CST. In addition, increased phosphorylation of AKT and FoxO1 implies improvement in insulin signaling by CST. Moreover, computational model predicted that enhanced phosphorylation of AKT along with reduction of pPERK can play an important role to enhance insulin sensitivity by alleviating ER stress.

In summary, this article, at first, demonstrates some *in vivo* experiments on NCD and DIO mice. Depending on the experimental patterns, a computational model based on state space equations is developed to integrate ER stress and insulin signaling pathways. Subsequently, we investigate whether simulation results follow the *in vivo* experimental results for both DIO (high ER stress) and NCD (normal) conditions. Next, the article shows the results on ER stress due to further *in vivo* experiments on DIO mice with CST treatment. Based on these experimental results, the computational model further explores the key markers to be targeted for achieving both alleviated ER stress and enhanced insulin sensitivity. The computational results are validated through *in vivo* experiments. In addition, *in silico* results predict the possible role of enhanced phosphorylation of AKT to increase insulin sensitivity overcoming high ER stress, which is verified in hepatocyte culture model where ER stress was induced by tunicamycin treatment but inhibited by AMG44. CST, like AMG44, inhibited PERK signaling and enhanced insulin effects.

## 2 Materials and methods

Here, we describe the experimental (*in vivo*) methodology first. Next, we elaborate how the computational model has been developed.

### 2.1 Animals, diets and treatments

**Fig. 2:**
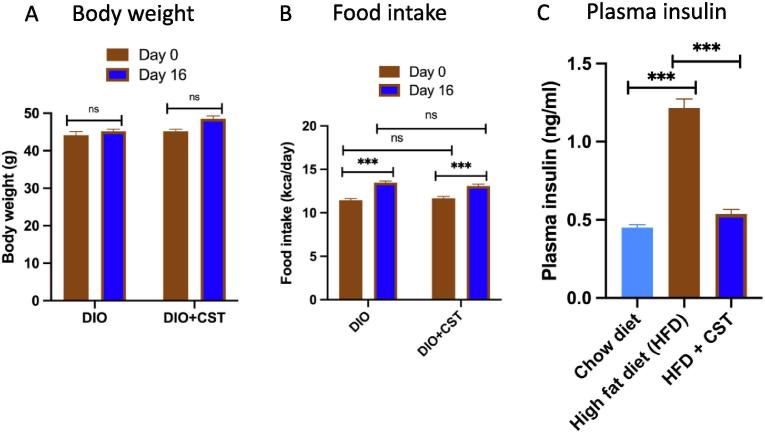
Body weights (A) were measured everyday, food intake (B) was measured on every other day by accounting the difference between initial food weights, and plasma insulin concentration (C) was determined on 16th day after collecting blood by snipping tail vein.

**Fig. 3:**
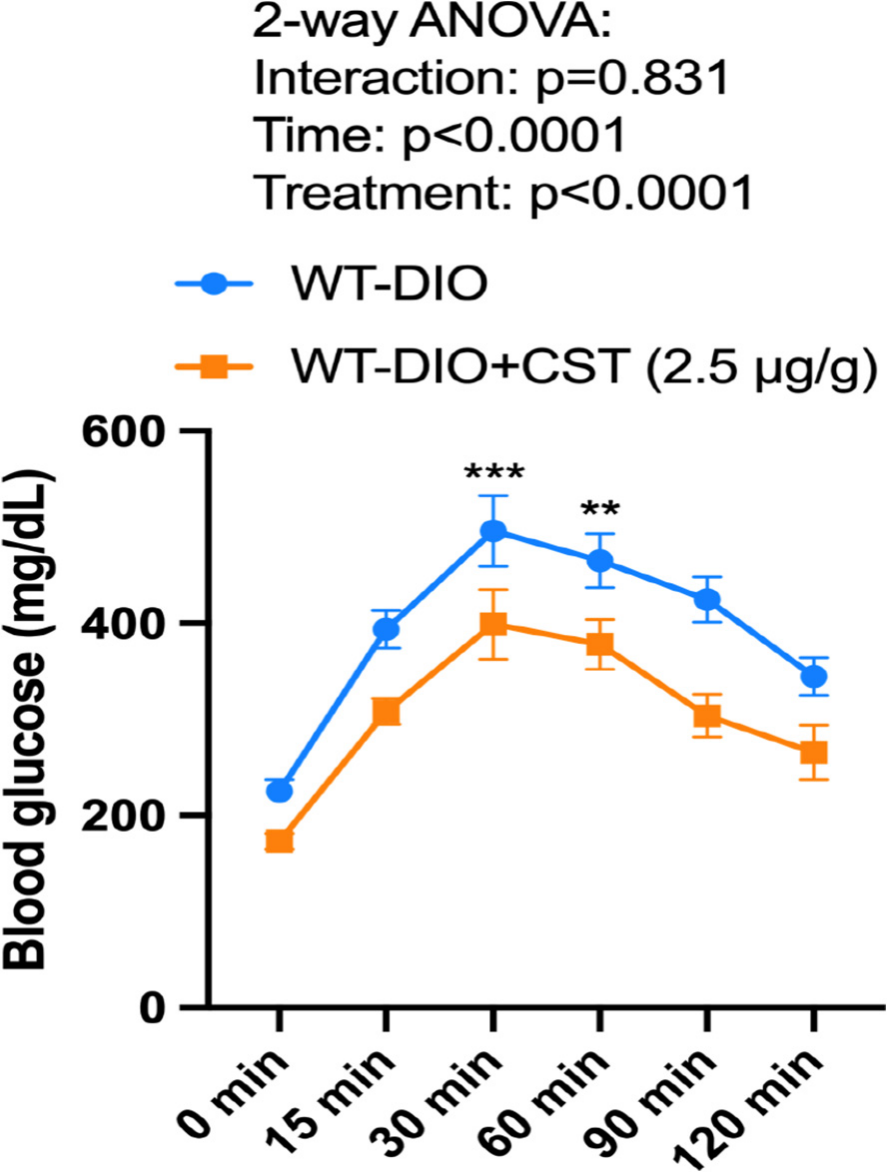
Oral glucose tolerance test (OGTT). A group of 8 DIO mice were injected IP with saline or CST (2.5 μg/g body weight/day) for 16 days. At zero minute, a drop of blood was collected from all 16 mice by snipping the tips of the tails and glucose concentration was measured by a monitor. Subsequently, glucose was gavaged into mice and tail blood was collected at various time points indicated and glucose concentration was measured.

DIO mice were created by feeding male wild-type (WT) mice (starting at 8 weeks of age) with a high-fat diet (HFD, Research diets D12492, 60% of calories from fat) for 16 weeks. Mice were kept in a 12:12 h dark/light cycle; food and water was available at all times. Control mice were fed an NCD (14% of calories from fat). Mice were treated with CST (2.5 μg/g BW IP for 15 days) after 11 weeks of HFD feeding when weight gains practically leveled off. In accordance with NIH animal care guidelines, all procedures and animals were housed and handled with the approval of The Institutional Animal Care and Utilization Committee. CST treatment did not alter body weight (Fig. 2A), food intake (Fig. 2B) but reduced plasma insulin levels (Fig. 2C). Oral glucose tolerance tests (OGTT) showed that CST treatment of DIO mice significantly reduced glucose intolerance (Fig. 3).

### 2.2 Hepatocyte isolation, culture and treatments

Male mice (16-week-old) were fed chow diet and used for perfusion of liver. Mice were perfused for 5 min with a calcium free buffer and followed by collagenase perfusion in a calcium containing buffer for another 5 min. Perfusion was carried out by inserting a catheter through inferior vena cave (IVC) and passing buffer through a tube and allowing buffer to come out through portal vein which was cut for this purpose. The procedure has been described in a published article [38]. Livers, after collagenase digestion, were excised out, hepatocytes were squeezed out in a petri dish inside a culture hood, filtered through 100-micron nylon filter, centrifuged at 50 x g for 5 min, and pellets were collected. The suspensions of cell pellets were then passed through 30% isotonic percoll by centrifuging at 100 x g for 10 min. Pellets were washed in buffer and suspended in culture medium (Williams E) containing glutamax, 10% FBS, 10 nM dexamethasone and antibiotics. Hepatocytes were seeded on collagen I coated plates. After 4 h of attachment, cultures were treated with tunicamycin (1μg/ml, Sigma), or CST (100 nM) or AMD44 (5 μM, TOCRIS) alone or in combination. Next day, after 12 h of incubation, cultures were exposed to serum-free medium for 4 h. Next, cultures were washed twice with Hepes-Krebs–Ringer-bicarbonate (HKRB) buffer and then incubated with or without insulin (10 nM) but containing the substrates, pyruvate (5 mM) and lactate (10 mM) for another 4 h. At the end of final incubation, culture media were collected for glucose assay using a commercial kit, and cultures were washed with PBS. Attached cells were dissolved in 1 M NaOH and subjected to protein assay. For western blotting, attached cells were dissolved in lysis buffer containing, detergent, protease inhibitor and phosphatase inhibitor cocktails (Sigma).

### 2.3 Transmission electron microscopy (TEM)

WT-NCD, WT-DIO and WT-DIO + CST livers were perfusion fixed through the left ventricle under deep anaesthesia. A pre-warmed (37°C) calcium and magnesium free buffer consisting of DPBS (Life Technologies Inc. Carlsbad, CA), 10 mM HEPES, 0.2 mM EGTA, 0.2% bovine serum albumin, 5 mM glucose and 10 mM KCl was used to flush mice for 3 min (3 ml per min; Langer Instruments Corp, Boonton, NJ). This is followed by perfusion with freshly prepared pre-warmed (37°C) fixative containing 2.5% glutaraldehyde, 2% paraformaldehyde in 0.15 M cacodylate buffer for 3 min. After dissecting, liver slices (2 mm thick) were put in the same fixative overnight (2 h at room temperature and 12 h at 4°C), and postfixed in 1% ${\mathrm{OsO}}_{4}$ in 0.1 M cacodylate buffer for 1 h on ice. Liver slices were stained *en bloc* with 2–3% uranyl acetate for 1 h on ice followed by dehydration in graded series of ethanol (20–100%) on ice, one wash with 100% ethanol and two washes with acetone (15 min each) and embedded with Durcupan. Approximately, 50 to 60 nm thick sections were cut on a Leica UCT ultramicrotome. Sections were picked upon Formvar and carbon-coated copper grids and stained with 2% uranyl acetate for 5 min and Sato’s lead stain for 1 min. Livers (3 from each group) were fixed and processed in two separate days. Stained grids were looked under a JEOL 1200EXII (JEOL, Peabody, MA) TEM and photographed with a Gatan digital camera (Gatan, Pleasanton, CA). Random micrographs were taken from 3 livers where samples were blinded. Also, 2 people did measurements randomly from different tissues as described previously [39]. The width of the ER lumen was determined by using the free-hand tool in NIH ImageJ 1.49 software as described by VLADENA KOUKALOVA (Mednet 2016, page722).

### 2.4 Immunoblotting

Homogenization of livers were made in a lysis buffer supplemented with phosphatase and protease inhibitors. Homogenates were subjected to 10% SDS-PAGE and immunoblotted. The following primary antibodies were obtained from Cell Signaling Technology (Boston, MA): AKT and pS473 AKT (both rabbit polyclonal), IRE (rabbit polyclonal 1:1000) and pS724-IRE (rabbit polyclonal 1:500), PERK (rabbit polyclonal 1:1000) and pT980-PERK (rabbit polyclonal 1:1000), eIF (rabbit polyclonal 1:1000) and pS51-eIF (rabbit polyclonal 1:500). Homogenates were immunoblotted for phospho-tyrosine using monoclonal pY antibody from Cell Signaling Technology, pY signals were identified as insulin receptor (IRpY) or insulin-receptor-substrate-1(IRS-1pY) by blotting the pY membranes with either anti-IR or anti-IRS-1 antibodies (also from Cell Signaling Technology).

### 2.5 Real Time PCR

RNeasy Mini Kit (Qiagen) was used to isolate total RNA from liver tissues. qScript cDNA synthesis kit (QuantaBio, Beverly, MA) was used to make the cDNAs, which were amplified by using PERFECTA SYBR FASTMIX L-ROX1250 (QuantaBio). The amplified samples were run on an Applied Biosystems 7500 Fast Real-Time PCR system (ABI). All PCRs were normalized to *Gapdh*, and relative expression levels were determined by the ΔΔ$C_{t}$ method. Primer sequences are provided in Table S1 of Supplementary File S1.

### 2.6 Computational analyses

The computational model involved formulation of state transition equations, input design, estimation of values for the kinetic parameters through model validation, and finally, application of PID controllers. In order to formulate state transition equations, we integrated ER stress and insulin signaling pathways as depicted in Fig. S1 of Supplementary File S1 using the method in one of our previous investigations [40]. Using these state transition equations together with estimated input and parameter values, the *in silico* state space model was developed. In order to investigate the significant markers to be targeted for higher insulin sensitivity along with alleviation of ER stress, we applied PID controllers on the state space model as depicted in Fig. S2 of Supplementary File S1.

#### 2.6.1 Formulation of state transition equations

Let us assume that the integrated biochemical pathway (Fig. S1 of Supplementary File S1) under investigation involves the state components $x_{1},x_{2},\ldots ,x_{n}$ representing different signaling molecules/proteins. Here, $u_{1}$ and $u_{2}$ are external inputs representing ER stress and insulin. Let $x_{1}$ is triggered by $u_{1}$. Here, $x_{1}$ is decayed/consumed at the rate proportional to $x_{1}\times x_{2}$. Thus, we can write,

(1) $${\mathrm{dx}}_{1}/\mathrm{dt}=k_{1}\times u_{1}-k_{2}\times x_{1}\times x_{2}$$

Here $k_{1}$ and $k_{2}$ are interaction rate and decay constants respectively.

Let us consider a situation as an example, where $x_{20}$ is activated by $x_{19}$ under the influence of $x_{1}$ and $x_{2}$. This activation is accelerated by $x_{18}$. Besides, $x_{20}$ is decayed/consumed at the rate proportional to $x_{20}$. Here, we can write,

(2) $${\mathrm{dx}}_{20}/\mathrm{dt}=k_{40}\times x_{19}\times (1+F_{2}\times x_{18})\times x_{1}\times x_{2}-k_{41}\times x_{20}$$

Here $k_{40}$ and $k_{41}$ are interaction rate and decay constants respectively, whereas $F_{2}$ is binding constant. Again, $x_{26}$ is triggered by $u_{2}$, and decayed/consumed at the rate proportional to $x_{26}$ along with other feedback effects due to $x_{30}$ and $x_{32}$. Thus, we can write,

(3) $${\mathrm{dx}}_{26}/\mathrm{dt}=k_{53}\times u_{2}-k_{52}\times x_{26}/(1+F_{3}\times x_{30})(1+F_{4}\times x_{32})$$

Here $k_{53}$ and $k_{52}$ are interaction rate and decay constants respectively, whereas $F_{3}$ and $F_{4}$ are binding constants. Similarly, we developed state transition equations for all other state components according to the biological phenomena. We have included all state transition equations in Supplementary File S1 to restrict the length of manuscript.

#### 2.6.2 Inputs $u_{1}$ and $u_{2}$

In order to get the equations for external inputs, we considered system equilibrium (steady state) condition. At this condition, we can say ${\mathrm{dx}}_{1}/\mathrm{dt}=0$ and ${\mathrm{dx}}_{26}/\mathrm{dt}=0$. Thus, we have

(4) $$u_{1}={\mathrm{amplify}}_{1}\times k_{2}\times x_{1}\times x_{2}/k_{1}$$

(5) $$u_{2}={\mathrm{amplify}}_{2}\times k_{52}/(1+F_{3}\times x_{30})(1+F_{4}\times x_{32})\times x_{26}/k_{53}$$

Here, ${\mathrm{amplify}}_{1}$ and ${\mathrm{amplify}}_{2}$ are constant terms. They are used to amplify the effect of external inputs to the system.

#### 2.6.3 Estimation of the values of the constant parameters through model validation

We solved the ordinary differential equations (ODEs), formulated above, with random values for the constant parameters (interaction rate, decay and binding constants) in [0, 5]. We initialized all state components at 1.05 and restrict their values in [1], [5] as depicted in Supplementary File S1. This ODE system was solved numerically (70000 iterations and in time span of [0 0.0002]) using ode23tb solver of Matlab software. As a result, the computational model was able to capture the behavioral pattern of different molecules (state components) under consideration at continuous time points. This is the advantage of ODE based model which is capable of calculating values of state components at continuous time points depending on the initial values provided in the beginning of simulation. The computational results were validated to check whether the model follows the experimental behavioral patterns or not for both DIO and NCD situations. Although the experimental western blot results provided molecular expression/concentration level (low/high) at single time point, they were quite capable of providing information about the change of molecular expression/concentration level during DIO and NCD situations. Based on this experimental knowledge-base, if the result did not follow the experimental patterns, we changed the values of the constant terms in an ad hoc manner to replicate the experimental behavior. Whenever the model followed the experimental behavioral patterns, we fixed the values of the constant parameters as provided in Supplementary File S1. Here we considered two situations - one calculating the value of $u_{1}$ (ER stress) using Eq. 4 resembling stress (DIO) and the other depicting $u_{1}$ (ER stress) = 0 as control (NCD).

#### 2.6.4 Application of PID controllers to investigate the significant markers to be targeted for alleviation of ER stress and enhanced insulin sensitivity

Here we applied two PID controllers on the ODE based model (state space model) considering it as a plant to test if the attenuation of ER stress resembling experimental CST effect and the enhancement of insulin sensitivity can be achieved simultaneously. In order to accomplish that the PID controllers were involved in controlling the signal levels of pPERK, IRpY, IRSpY and pAKT. Here we used Simulink platform of Matlab software for simulation. Depending on error functions, appropriately calculated control (external) inputs for ER stress and insulin were applied on the state space model. The general form of the error function can be defined as

(6) e(t)=|referenceinput(r(t))-correspondingoutput(o(t))|

Finally we considered the tuned parameter values as $k_{p}$ = 0, $k_{i}$ = 0.0691 and $k_{d}$ = 0 for first PID controller. While for the second PID controller, we tuned parameter values as $k_{p}$ = 0.2134, $k_{i}$ = 0.10329 and $k_{d}$ = −0.1082. Here $k_{p},k_{i}$ and $k_{d}$ represent proportional, integral and derivative constants respectively. The general form of the control (external) input function (u(t)) can be defined as

(7) $$u(t)=k_{p}\times e(t)+k_{i}\int_{0}^{t}e(t^{\prime }){\mathrm{dt}}^{\prime }+k_{d}\times \mathrm{de}(t)/\mathrm{dt}$$

## 3 Results

In this section, at first, we demonstrate how computational model of integrated ER stress and insulin signaling pathway successfully mimics the experimental (*in vivo*) behavior of DIO (with high ER stress) and NCD mice. Thereafter, some more results from computational model depict the effects of high ER stress on insulin signaling pathway. Next, the *in vivo* results on DIO mice with CST treatment are illustrated. Then, we computationally explore the significant markers to be targeted for higher insulin sensitivity with alleviation of ER stress using some “thought experiments”. Finally, the computational results are validated through *in vivo* experiments as well as in hepatocyte culture model.

### 3.1 Hepatocyte ER dilation and activation of ATF6 branch of UPR pathway

**Fig. 4:**
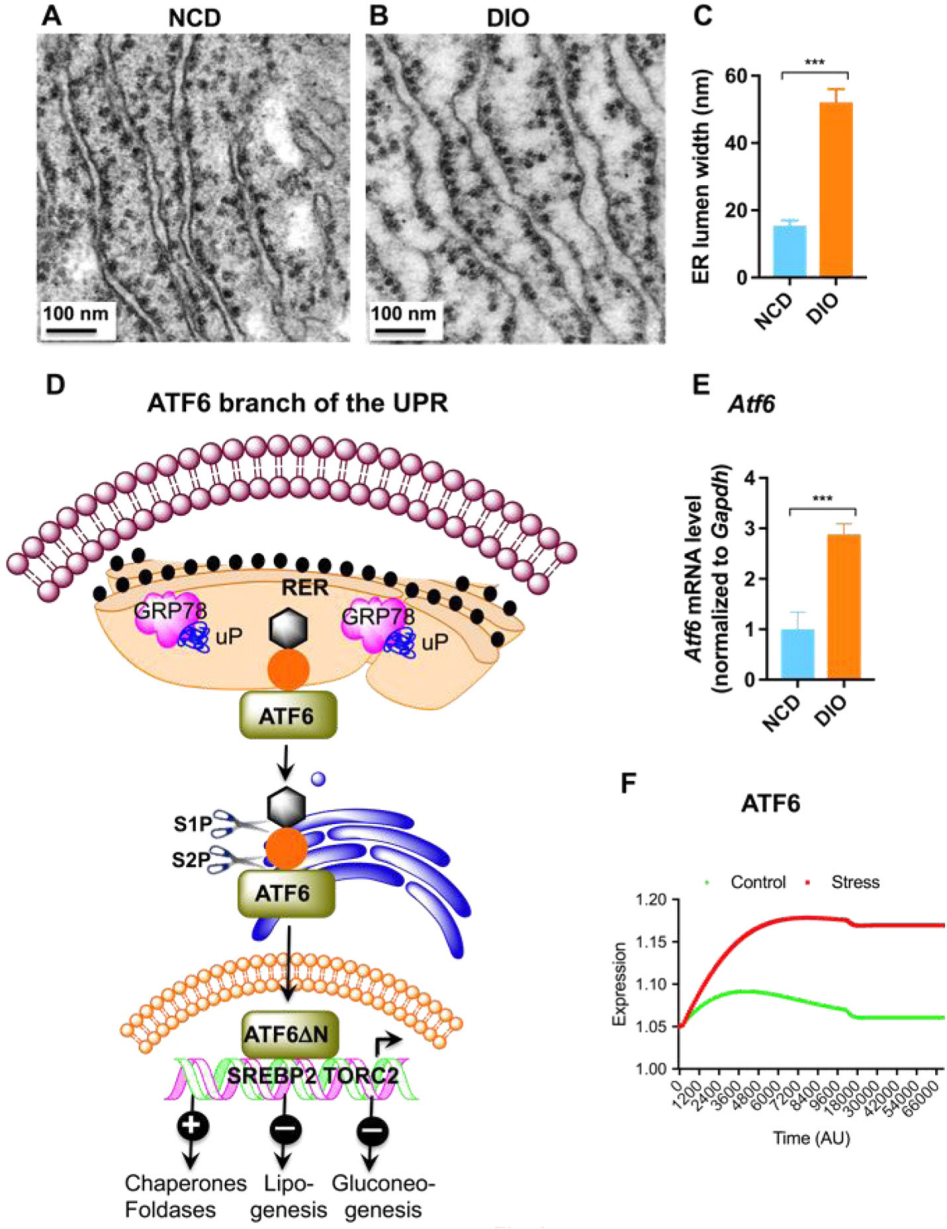
(A & B) TEM photographs showing dilation of ER lumen in ultrathin (∼60 nm) liver sections of DIO mice. (C) Morphometric analyses of ER lumen width in NCD and DIO mice. (D) Schematic diagram showing the ATF branch of the UPR. (E) Changes in ATF6α mRNA levels in NCD and DIO mice (n = 6). (F) *In silico* state space model resembling the behavior of ATF6α with the experimental results. The expression of ATF6α is higher during stress (DIO) condition than that in control (NCD).

The increased demand on the synthetic machinery during obesity results in unfolded or misfolded proteins accumulation in the ER lumen leading to ER stress, activating the UPR. Our ultrastructural studies show dilated ER lumen possibly to accommodate increased unfolded/misfolded proteins (Fig. 4A–C). ATF6α is an ER type-II transmembrane protein harbouring a bZIP transcription factor on its cytosolic domain and a C-terminal luminal region that senses ER stress (Fig. 4D). To counteract with the ER stress, ATF6α transits to the Golgi apparatus for its cleavage by the proteases S1P and S2P, which releases the cytosolic domain (ATF6f) [41]. ER’s capacity for folding is increased as a result of ATF6f-induced expression of protein chaperone genes including ER protein 57 (ERp57), binding immunoglobulin protein (BiP or GRP78) and glucose-regulated protein (GRP) 74. ATF6f also decreases lipogenesis in liver by antagonizing SREBP2 [42] and inhibits gluconeogenesis by interacting with CRTC2 [43] or inhibition of CREB [44]. Consistent with the existing literature, we found increased ATF6α mRNA level in DIO liver [45], [46] (Fig. 4E).

Depending on the aforesaid experimental results, we validated the *in silico* state space model during stress (DIO) and control (NCD) conditions. We found higher expression of ATF6α during stress (DIO) than that in control (NCD) after 70000 iterations (Fig. 4F), which corresponds to increased ATF6α mRNA in DIO liver (Fig. 4E).

### 3.2 Activation of IRE1α branch of UPR

**Fig. 5:**
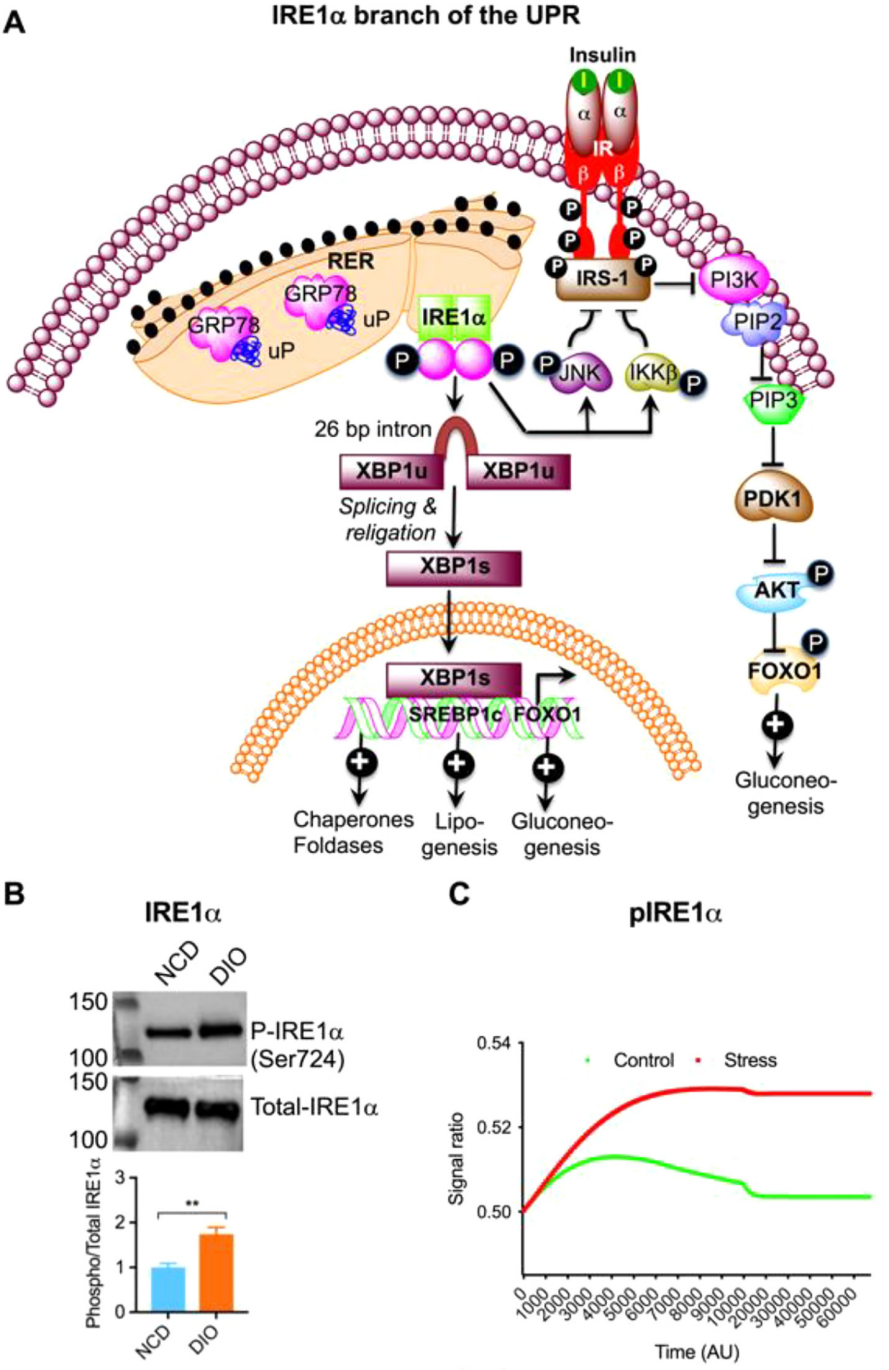
(A) Schematic diagram showing the IREα branch of the UPR. (B) Western blots showing increased phosphorylation of IRE1α in DIO liver (n = 4). (C) *In silico* state space model resembling the behavior of pIRE1α with the experimental results. The ratio (pIRE1α/IRE1α) is higher during stress (DIO) condition than that in control (NCD).

The transmembrane protein IRE1α contains an N-terminal luminal sensor domain, a C-terminal cytosolic effector, and a single transmembrane domain harbouring a Ser/Thir kinase domain and an endoribonuclease domain (Fig. 5A). ER stress induces dimerization and trans-autophosphorylation of IRE1α, thereby causing an induction of a conformational change and the subsequent activation of its RNAse domain to selectively cleave a 26-nucleotide intron within the XBP1 mRNA [47], [48]. This unconventional splicing introduces a translational frameshift to generate a stable and active transcription factor XBP1s. Active XBP1s regulate the expression of genes that modulate protein folding, secretion, translocation, ERAD into the ER, and synthesis of lipid [49], [50]. To alleviate the protein-folding load on the ER, the RNAse domain of IRE1α also modulates the “regulated IRE1-dependent decay (RIDD)” [51]. IRE1α also induces lipogenesis [52], [53] and gluconeogenesis [54]. Consistent with the existing literature, we found increased phosphorylation of IRE1α (pIRE1α) in DIO liver (Fig. 5B). The state space model also showed higher signal of pIRE1α during stress (DIO) than that in control (NCD) (Fig. 5C), which corresponds to increased phosphorylated IRE1α in DIO liver (Fig. 5B).

### 3.3 Activation of PERK branch of UPR

**Fig. 6:**
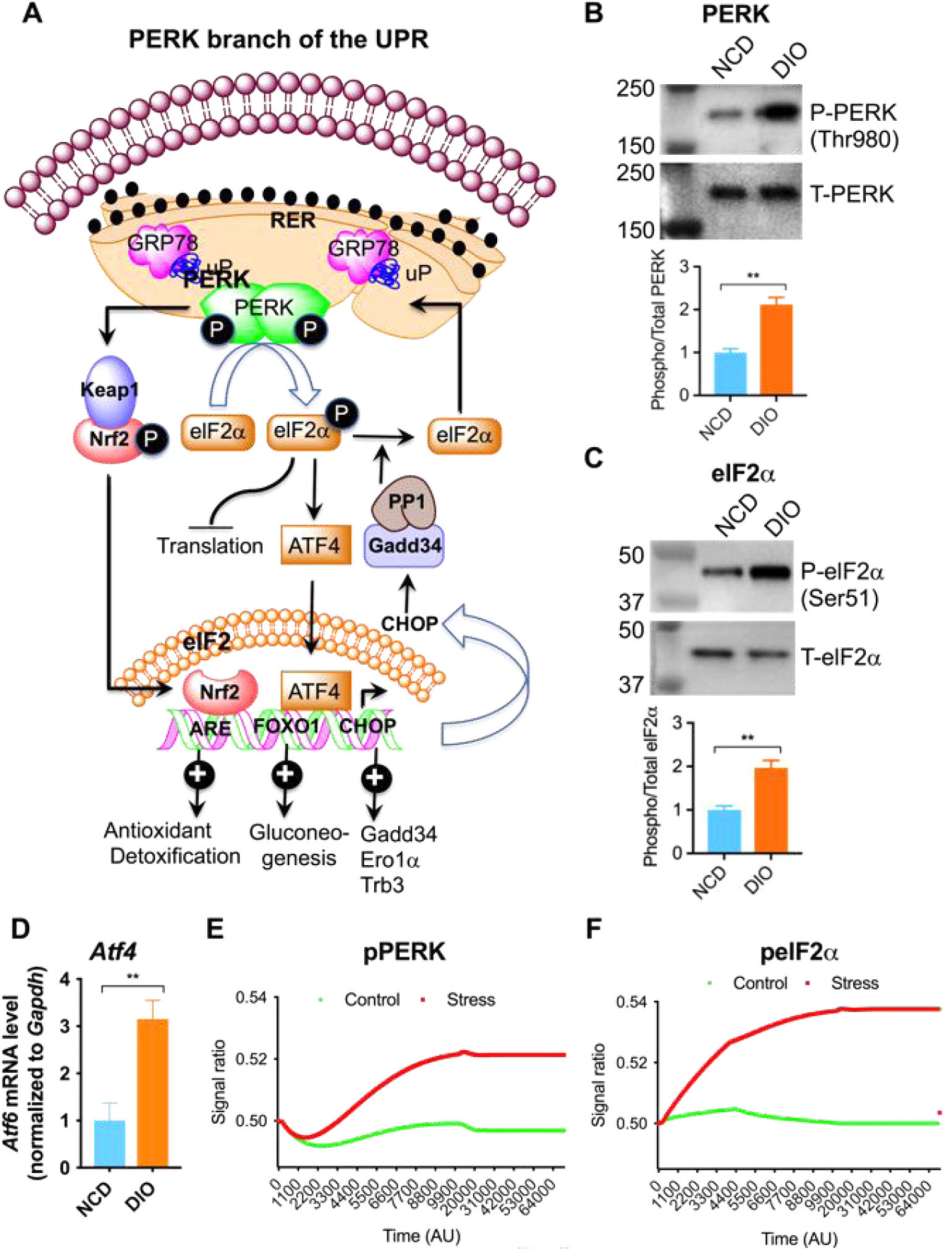
(A) Schematic diagram showing the PERK branch of the UPR. (B) Western blots showing increased phosphorylation of PERK at Thr980 in DIO liver (n = 4). (C) Western blots showing increased phosphorylation of elF2α at Ser51 in DIO liver (n = 4). (D) Changes in ATF4 mRNA level in NCD and DIO liver (n = 6). (E & F) *In silico* state space model resembling the behavior of pPERK and peIF2α with the experimental results. The ratios (pPERK/PERK) and (pelF2α/elF2α) are higher during stress (DIO) condition than that in control (NCD).

Phosphorylation at Thr980 activates PERK to alleviate the protein-folding load on the ER. Phosphorylated PERK phosphorylates eIF2α at Ser51 to briefly halt the initiation of mRNA translation. This leads to the reduction of global protein synthesis resulting in decreased workload of the ER (Fig. 6A) [55], [56]. Paradoxically phosphorylated eIF2α (peIF2α) also up regulates the transcription and translation of many mRNAs, such as nuclear erythroid 2 p45-related factor 2 (Nrf2), activating transcription factor-4 (ATF4), and nuclear factor kappa b (NF-κB). ATF4, produced through alternative translation, influences gene expressions involved in ER redox control (ERO1, ER oxidoreductin), apoptosis (CHOP, C/EBP homologous protein), glucose metabolism (fructose 1,6-bisphosphate; glucokinase, and phosphoenolpyruvate carboxykinase), and the negative feedback release of eIF2α inhibition (Gadd34, growth arrest, and DNA damage-inducible protein) (Fig. 6A) [57], [58]. Consistent with the above literature, we found increased phosphorylation of PERK at Thr980 (Fig. 6B) and eIF2α at Ser51 (Fig. 6C) as well as increased mRNA level of ATF4 (Fig. 6D).

The *in silico* state space model was quite effective to mimic the aforesaid experimental results. The model showed higher signals of pPERK (Fig. 6E) and peIF2α (Fig. 6F) during stress (DIO) than that in control (NCD), which corresponds to increased phosphorylated PERK (Fig. 6B) and phosphorylated eIF2α (Fig. 6C) in DIO liver.

### 3.4 Computational model showing stress (HFD)-changes in insulin and inflammatory signaling

**Fig. 7:**
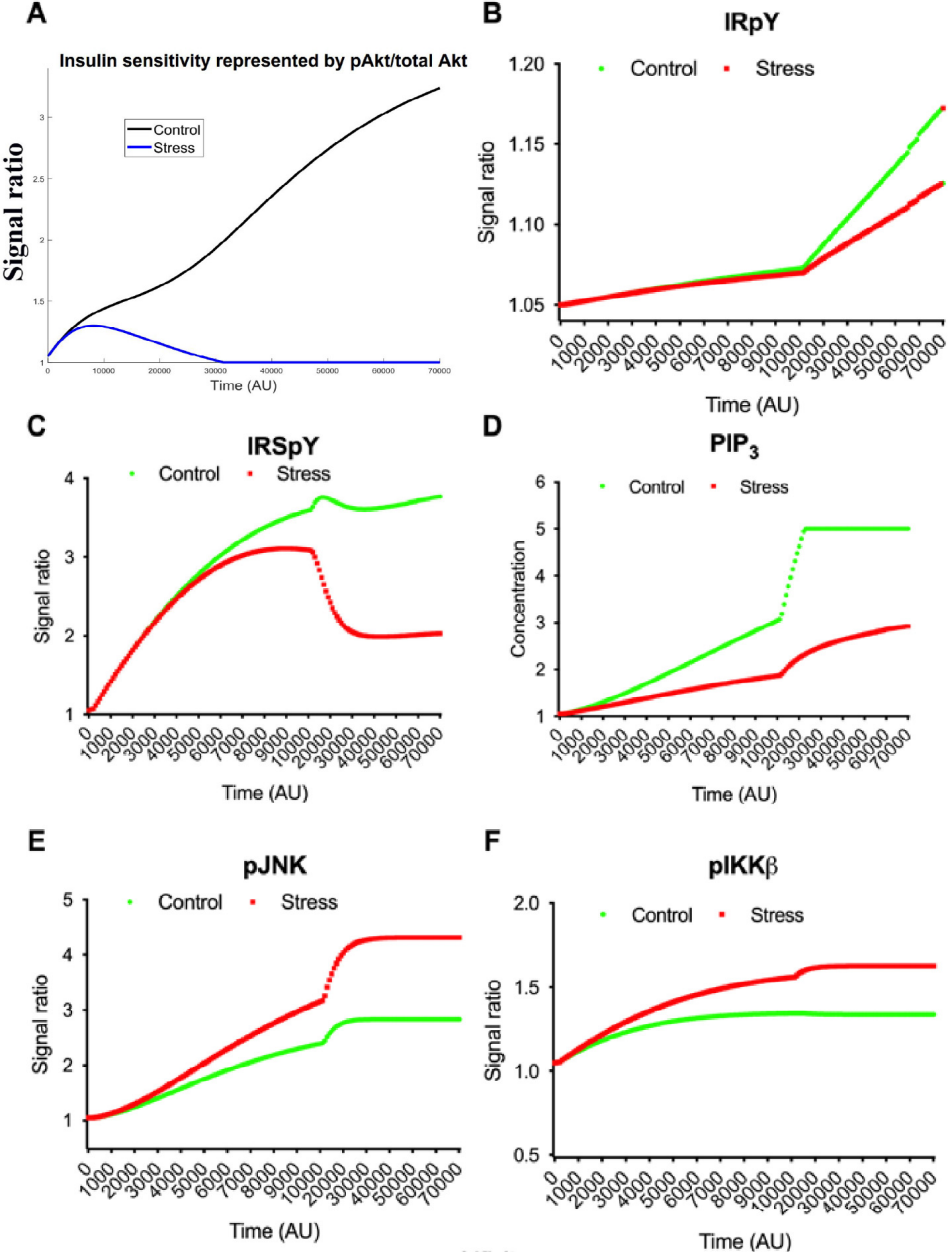
*In silico* state space model depicting that (A) insulin sensitivity (represented by pAkt/total Akt signal ratio) is higher in control (NCD) than that in stress (DIO). Besides, signals of phosphorylated forms of intermediate molecules (B) IRpY, (C) IRSpY and (D) concentration of ${\mathrm{PIP}}_{3}$ enhance in control (NCD) in comparison with stress (DIO) condition. On the other hand, the expressions of (E) pJNK and (F) pIKKβ become higher during stress (DIO) than in control (NCD) scenario.

Previous investigations [59], [60] showed that ER stress could lead to insulin resistance through different ways including activation of pJNK and pIκB kinase β (pIKKβ). In line with the existing literature, the *in silico* state space model yielded the following: enhanced insulin sensitivity (represented by pAkt/total Akt signal ratio) in control (NCD) compared to that under stress (DIO) (Fig. 7A); increased levels of IRpY (Fig. 7B), IRSpY (Fig. 7C), phosphorylated Forkhead box protein O1 (pFoxO1) (Fig. S3A of Supplementary File S1) and increased concentration of phosphatidylinositol trisphosphate (${\mathrm{PIP}}_{3}$) (Fig. 7D) in control (NCD) compared to that under stress (DIO) condition. On the other hand, the signal levels of pJNK (Fig. 7E), pIKKβ (Fig. 7E), and pNF-κB (Fig. S3B of Supplementary File S1) were higher during stress (DIO) than that in control (NCD) scenario.

### 3.5 Decreased ER stress due to CST treatment

**Fig. 8:**
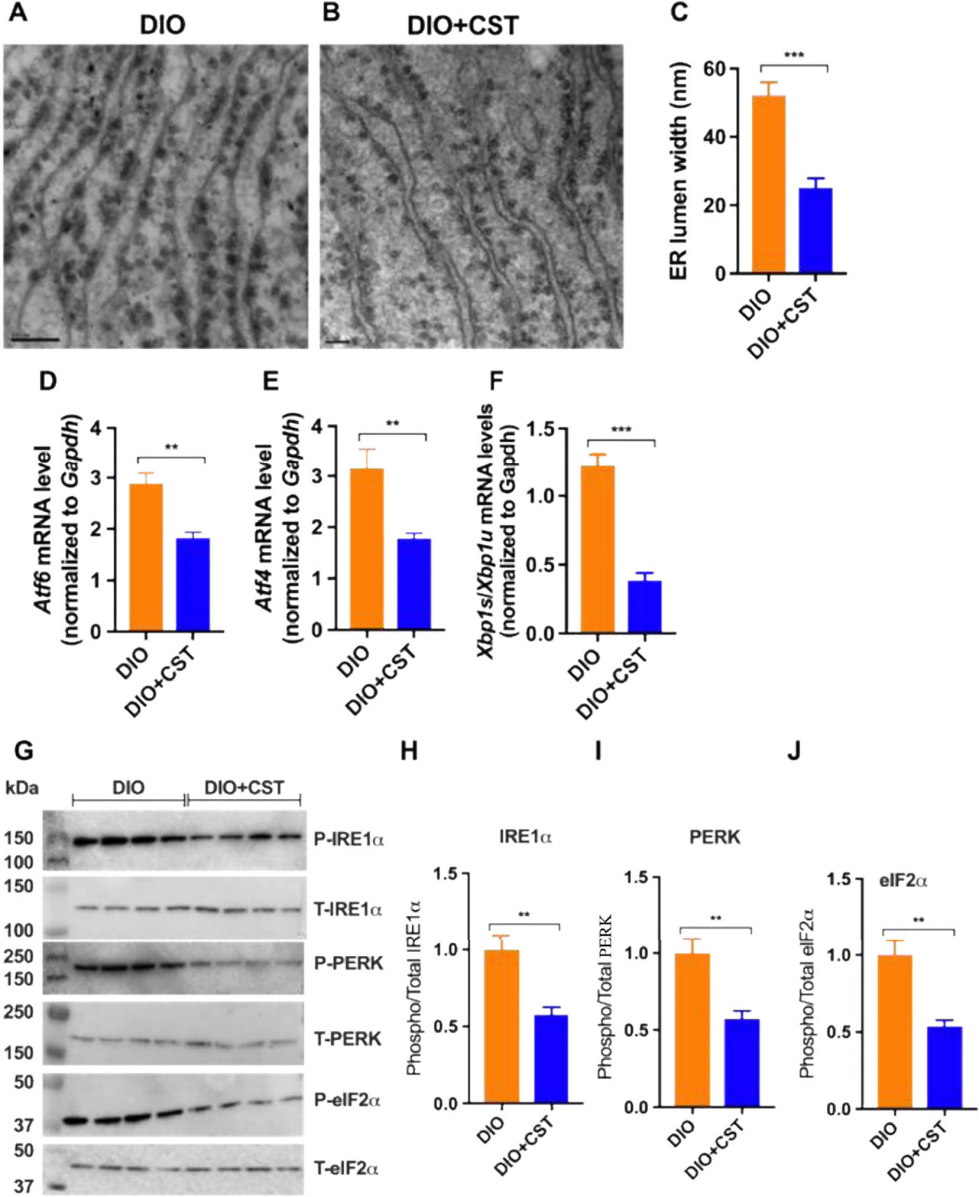
(A & B) TEM photographs showing attenuation of ER lumen in DIO liver sections after treatment with CST. (C) Morphometric analyses of TEM photographs showing decreased ER lumen width after treatment with CST. qPCR analyses showing CST-induced decrease in mRNA levels of (D) ATF6α, (E) ATF4, and (F) ratio between spliced versus unspliced Xbp1 in DIO liver. (G) Western blots showing decreased phosphorylation of UPR signaling molecules in DIO liver after treatment with CST: (I) phosphorylated PERK/total PERK, (J) phosphorylated elF2α/total elF2α, and (H) phosphorylated IRE1α/total IRE1α.

In lean state, adipose tissue macrophages (ATM) and resident macrophages (Kupffer cells) in liver exhibit an anti-inflammatory alternatively activated (M2) phenotype [61], [62], [63], [64], [65]. Complex molecular interactions between diet, environment and genetics at the metabolic tissues (adipocytes, heaptocytes, and pancreatic islets) and immune system (macrophages, neutrophils, and lymphocytes) provoke a low-grade, chronic inflammatory response, which is called metaflammation [66]. Obesity, characterized by metaflammation [67], [68], [69], is allied with ER stress that disrupts glucose homeostasis [2], [20], [70] and results in the development of atherosclerotic plaques [71], [72], [73]. In obesity, ATMs and Kupffer cells display a predominantly proinflammatory classically activated (M1) phenotype, which is thought to promote insulin resistance and type 2 diabetes [61], [74], [75], [76], [77], [78], [79]. It has been recently shown that IRE1α mediates saturated fatty acid-induced activation of the NLRp3 inflammasome in human and mouse macrophages [80] and that macrophage-specific deletion of IRE1α conferred resistance to high-fat diet-induced obesity, thereby linking macrophages to ER stress, metaflammation and insulin sensitivity [81]. We have recently shown that CST improves insulin sensitivity by inhibiting obesity-induced inflammation and macrophage infiltration in the liver and by suppressing glucose production in hepatocyte [33]. Therefore, we reasoned that CST would decrease obesity-induced ER stress. Consistent with our hypothesis we found that CST treatment exhibits ER stress lowering effects: (i) decrease of obesity-induced ER dilation (Fig. 8A-C) decreased mRNA abundance for ATF6 (Fig. 8D and ATF4 genes (Fig. 8E, (iii) decreased abundance of (Fig. 8F) (iv) spliced Xbp1 mRNA decreased phosphorylation of PERK (Fig. 8G & I), eIF2α (Fig. 8G & J), and IRE1α (Fig. 8G & H).

### 3.6 Application of PID controller exploring markers to be targeted for higher insulin sensitivity with reduced ER stress

We observed that the state space model successfully mimicked the cellular behavior during both DIO and NCD conditions in consistent with the experimental results and existing literature. In addition, the aforesaid experimental results confirmed that CST alleviated ER stress. Literature showed ER stress would contribute to insulin resistance. Thus it indirectly implies that attenuation of ER stress in hepatic macrophages by CST may be an additional mechanism to enhance insulin sensitivity. In order to test the hypothesis, we applied two PID controllers on the state space model targeting pPERK, IRpY, IRSpY and pAKT to a high or low value to explore a significant marker or a combination of significant markers to be targeted for enhancing insulin sensitivity with reduction of ER stress. It was clear from our aforementioned *in vivo* experiments that pPERK was a significant target-marker of CST to alleviate ER stress. However, our *in vivo* experiments did not confirm whether targeting only pPERK was enough to enhance insulin sensitivity along with alleviation of ER stress. In order to verify it, we considered following three cases targeting (I) pPERK and IRpY, (II) pPERK and IRSpY and (III) pPERK and pAKT as “thought experiments”.

#### 3.6.1 Case I (Targeting pPERK and IRpY)

Here we checked all possible four conditions - (i) High pPERK and low IRpY, (ii) High pPERK and high IRpY, (iii) Low pPERK and high IRpY and (iv) low pPERK and low IRpY.

**Fig. 9:**
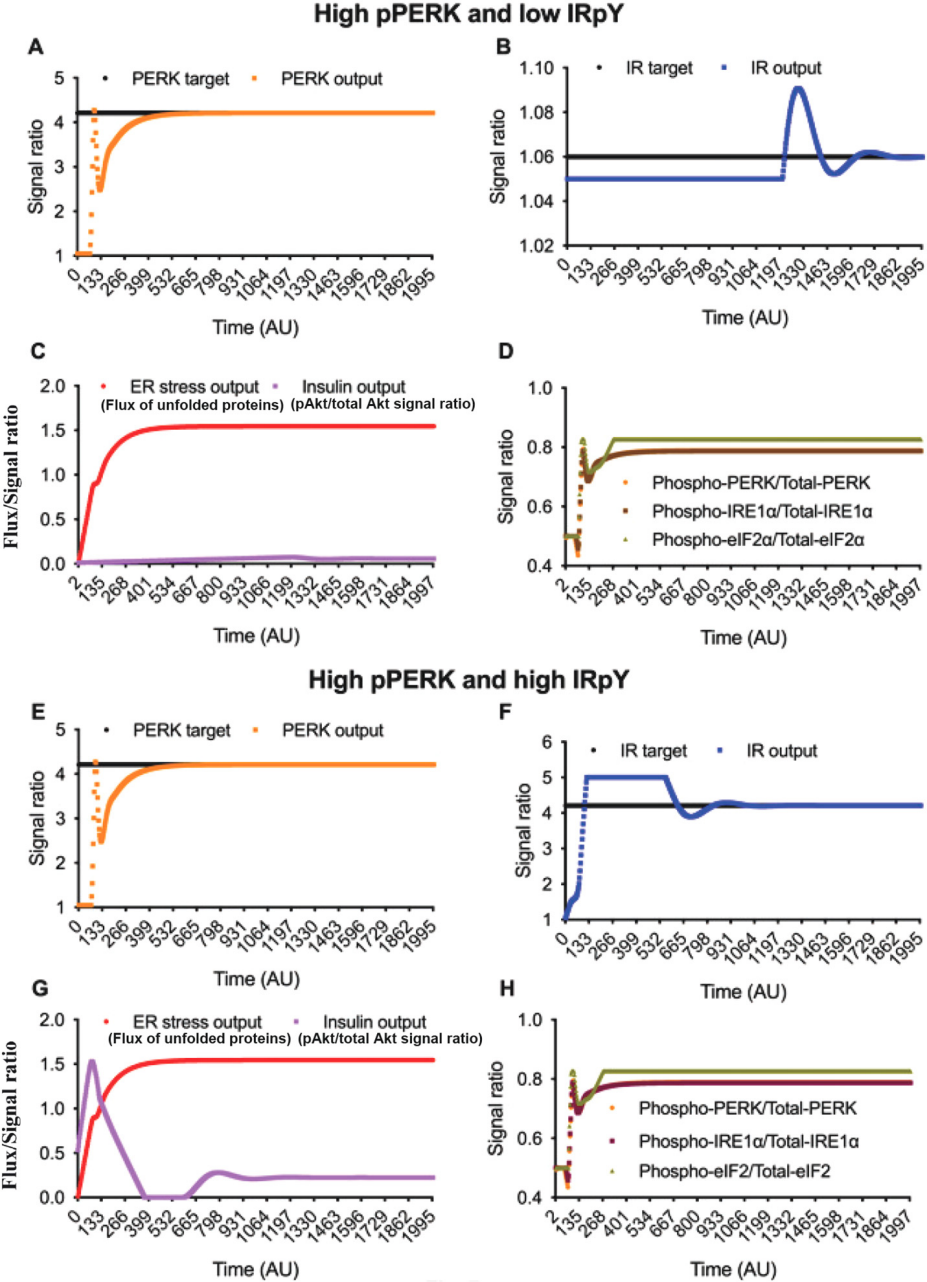
**High pPERK and low IRpY.** Here (A) phosphorylated PERK output as well as (B) tyrosine phosphorylated IR output is controlled by the Proportional-Integral-Derivative (PID) controller according to the reference input PERK target and IR target respectively. As a result, (C) shows high ER stress (flux of unfolded proteins) and low insulin sensitivity (pAkt/total Akt signal ratio). Besides, (D) ratios (phosphorylated PERK/total-PERK), (phosphorylated IRE1α/total IRE1α) and (phosphorylated elF2α/total-elF2α) are quite high around 0.8. **High pPERK and high IRpY.** Here (E) phosphorylated PERK output as well as (F) tyrosine phosphorylated IR output is controlled by Proportional-Integral-Derivative (PID) controller according to the reference input PERK target and IR target respectively. As a result, (G) shows high ER stress (flux of unfolded proteins) and low insulin sensitivity (pAkt/total Akt signal ratio). Besides, (H) ratios (phosphorylated PERK/total PERK), (phosphorylated IRE1α/total IRE1α) and (phosphorylated eIF2α/total eIF2α) are quite high around 0.8.
(ii) **High pPERK and high IRpY**
  In this condition, both signals of pPERK and IRpY were set to high values (Fig. 9E and F). We observed that high signal of IRpY was not able to raise insulin sensitivity as ER stress became high (Fig. 9G). Subsequently, the other ratios (pPERK/PERK), (pIRE1α/IRE1α) and (peIF2α/eIF2α) became high (Fig. 9H). It is clear that when pPERK is high, i.e., the ratios are high, insulin sensitivity cannot be high in spite of high IRpY signal. In this context, it should be mentioned that this condition is naturally not possible because ER stress (PERK signaling) and Insulin signaling are inversely related. Both cannot be high at the same time. However, an artificial situation can be created where DIO mice are infected with virus expressing a mutant IR that mimics pY. But such mice are not available.
(iii) **Low pPERK and high IRpY**
  For this condition, the signal value of pPERK was targeted to be low while IRpY to be high (Fig. 10A and B). In this context, experimental results demonstrated CST alleviated ER stress (Fig. 8A–C and 11A–G), which led to decreased pPERK (Fig. 8G & I), peIF2α (Fig. 8G & J) and pIRE1α (Fig. 8G & H). Similarly, this case depicted low ER stress (Fig. 10C) along with low values for the ratios (pPERK/PERK), (pIRE1α/IRE1α) and (peIF2α/eIF2α) (Fig. 10D). In addition, we noticed high insulin sensitivity (Fig. 10C) here. The *in vivo* verification (Fig. 15) of this condition will be discussed in following subsections.

(i) **High pPERK and low IRpY**
  Here we set a high signal value for pPERK and a low signal value for IRpY (Fig. 9A and B). In this context, experimental results demonstrated that ER stress increased pPERK (Fig. 6B), peIF2α (Fig. 6C), and pIRE1α (Fig. 5B). In addition, the state space model showed decreased insulin sensitivity (Fig. 7A) and IRpY signal (Fig. 7B) in ER stress. Consistent with these results, this condition depicted high ER stress (represented by the flux of unfolded proteins) and low insulin sensitivity (represented by pAkt/total Akt signal ratio) along with high values for the ratios, i.e., (pPERK/PERK), (pIRE1α/IRE1α) and (peIF2α/eIF2α) (Fig. 9C and D).

**Fig. 10:**
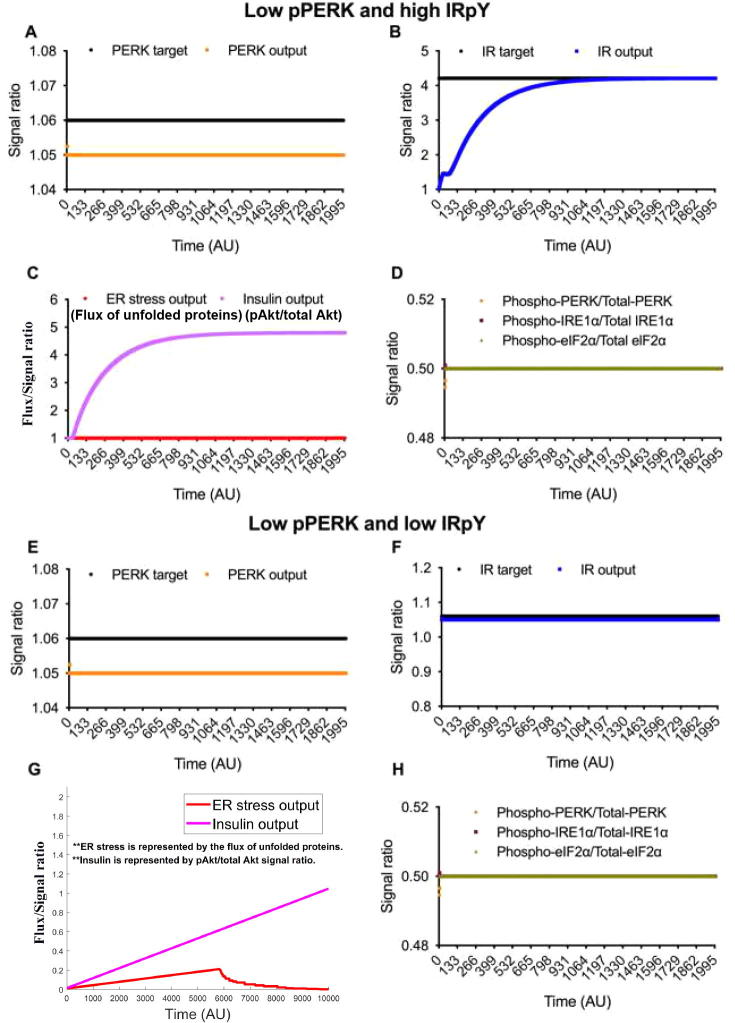
**Low pPERK and high IRpY.** Here (A) phosphorylated PERK output as well as (B) tyrosine phosphorylated IR output is controlled by the Proportional-Integral-Derivative (PID) controller according to the reference input PERK target and IR target respectively. As a result, (C) shows low ER stress (flux of unfolded proteins) and high insulin sensitivity (pAkt/total Akt signal ratio). Besides, (D) ratios (phosphorylated PERK/total PERK), (phosphorylated IRE1α/total IRE1α) and (phosphorylated eIF2α/total eIF2α) are quite low around 0.5. **Low pPERK and low IRpY.** Here (E) phosphorylated PERK output as well as (F) tyrosine phosphorylated IR output is controlled by the Proportional-Integral-Derivative (PID) controller according to the reference input PERK target and IR target respectively. As a result, (G) shows that ER stress (flux of unfolded proteins) is very low, while insulin sensitivity (pAkt/total Akt signal ratio) is increasing. Besides, (H) ratios (phosphorylated PERK/total PERK), (phosphorylated IRE1α/total IRE1α) and (phosphorylated eIF2α/Total eIF2α) are quite low around 0.5.

**Fig. 11:**
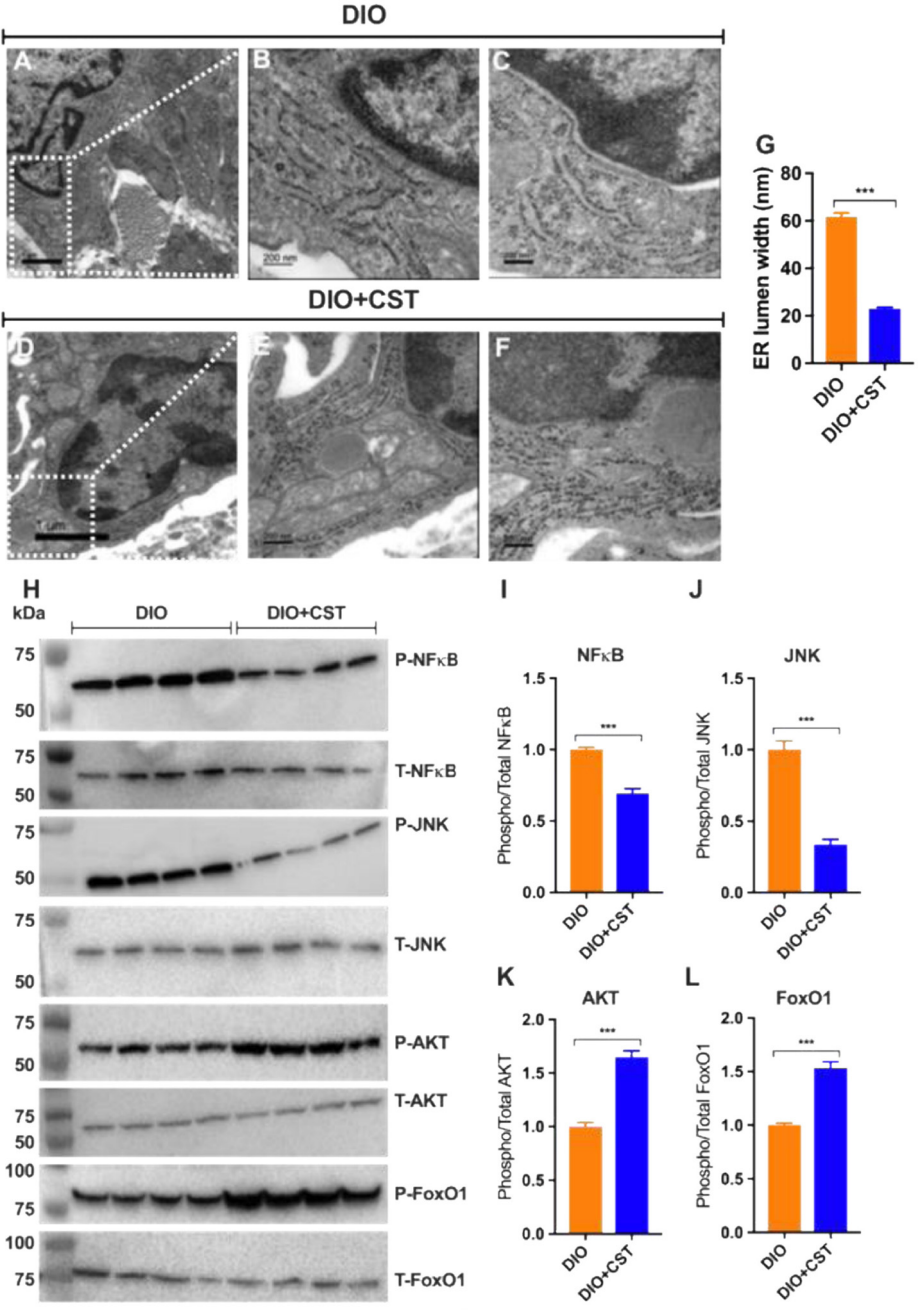
TEM photographs showing ER in infiltrated macrophages in DIO liver sections after treatment with CST. (A) Low magnification and (B & C) high magnification micrographs showing ER dilation in DIO mice. (D) Low magnification and (E & F) high magnification micrographs showing ER dilation in DIO mice treated with CST. (G) Morphometric analyses of ER lumen diameter in DIO and DIO + CST-treated mice. Western blots showing (H) decreased phosphorylation of NF-κB and (H) JNK coupled with (H) increased phosphorylation of AKT and FoxO1 in DIO liver after treatment with CST. The corresponding densitometric values are shown as follows: (I) NF-κB, (J) JNK, (K) AKT, and (L) FoxO1.
(iv) **Low pPERK and low IRpY**
  Here, we set low signal values for both pPERK and IRpY (Fig. 10E and F). Here, we observed very low ER stress (Fig. 10G) along with low values for the ratios (pPERK/PERK), (pIRE1α/IRE1α) and (peIF2α/eIF2α) (Fig. 10H). We also observed that insulin sensitivity had been increasing slowly because of low IRpY signal, whereas ER stress output (the flux of unfolded proteins caused by ER stress) dropped to almost zero after near time 6000 AU. (Fig. 10G). This condition may correspond to a situation where CST works in an insulin-independent way. Here CST is suppressing JNK and NFkB signaling, macrophage-induced inflammation and indirectly helping insulin signaling.

From the above four conditions, we clearly observed enhanced insulin sensitivity by reducing pPERK which can be achieved experimentally by applying CST on DIO mice. Besides, low ER stress and low values of the ratios (pPERK/PERK), (pIRE1α/IRE1α) and (peIF2α/eIF2α)) would result in high insulin sensitivity. Thus we can conclude that CST not only reduces ER stress but also increases insulin sensitivity.

#### 3.6.2 Case II (Targeting pPERK and IRSpY)

Similar to case I, we checked all possible four conditions - (i) High pPERK and low IRSpY, (ii) High pPERK and high IRSpY, (iii) Low pPERK and high IRSpY and (iv) low pPERK and low IRSpY.

(i) **High pPERK and low IRSpY**
  Here a high signal value for pPERK and a low signal value for IRSpY (Fig. S4A-B of Supplementary File S1) led to high ER stress and low insulin sensitivity along with high values for the ratios, i.e., (pPERK/PERK), (pIRE1α/IRE1α) and (peIF2α/eIF2α) (Fig. S4C-D of Supplementary File S1).
(ii) **High pPERK and high IRSpY**
  High signal of IRSpY (Fig. S4G of Supplementary File S1) was not able to raise insulin sensitivity (Fig. S4G of Supplementary File S1) because of high pPERK (Fig. S4E of Supplementary File S1) resulting in high ER stress (Fig. S4G of Supplementary File S1). Subsequently, the other ratios (pPERK/PERK), (pIRE1α/IRE1α) and (peIF2α/eIF2α) became high (Fig. S4H of Supplementary File S1). As mentioned earlier, ER stress (PERK signaling) and Insulin signaling are inversely related. Both cannot be high at the same time. Thus, this condition is naturally not possible.
(iii) **Low pPERK and high IRSpY**
  When the signal value of pPERK was targeted to be low while IRSpY to be high (Fig. S5A-B of Supplementary File S1), we noticed high insulin sensitivity (Fig. S5C of Supplementary File S1). Besides, it depicted low ER stress (Fig. S5C of Supplementary File S1) along with low values for the ratios (pPERK/PERK), (pIRE1α/IRE1α) and (peIF2α/eIF2α) (Fig. S5D of Supplementary File S1). This situation arises when CST is applied as per our experimental verification (Fig. 15) discussed later.
(iv) **Low pPERK and low IRSpY** Similarly, low signal value for pPERK (Fig. S5E of Supplementary File S1) led to increasing insulin sensitivity (Fig. S5G of Supplementary File S1). However, insulin sensitivity had been increasing slowly because of low IRSpY signal (Fig. S5F of Supplementary File S1). Besides, we observed very low ER stress (Fig. S5G of Supplementary File S1) compared to DIO situation along with low values for the ratios (pPERK/PERK), (pIRE1α/IRE1α) and (peIF2α/eIF2α) (Fig. S5H of Supplementary File S1). As mentioned earlier, this condition may arise if CST works in an insulin-independent way.

Thus, reduction of pPERK on application of CST is the main factor to enhance insulin sensitivity.

#### 3.6.3 Case III (Targeting pPERK and pAKT)

Similar to previous two cases, we investigated all possible four conditions – (i) High pPERK and low pAKT, (ii) High pPERK and high pAKT, (iii) Low pPERK and high pAKT (iv) Low pPERK and low pAKT.

**Fig. 12:**
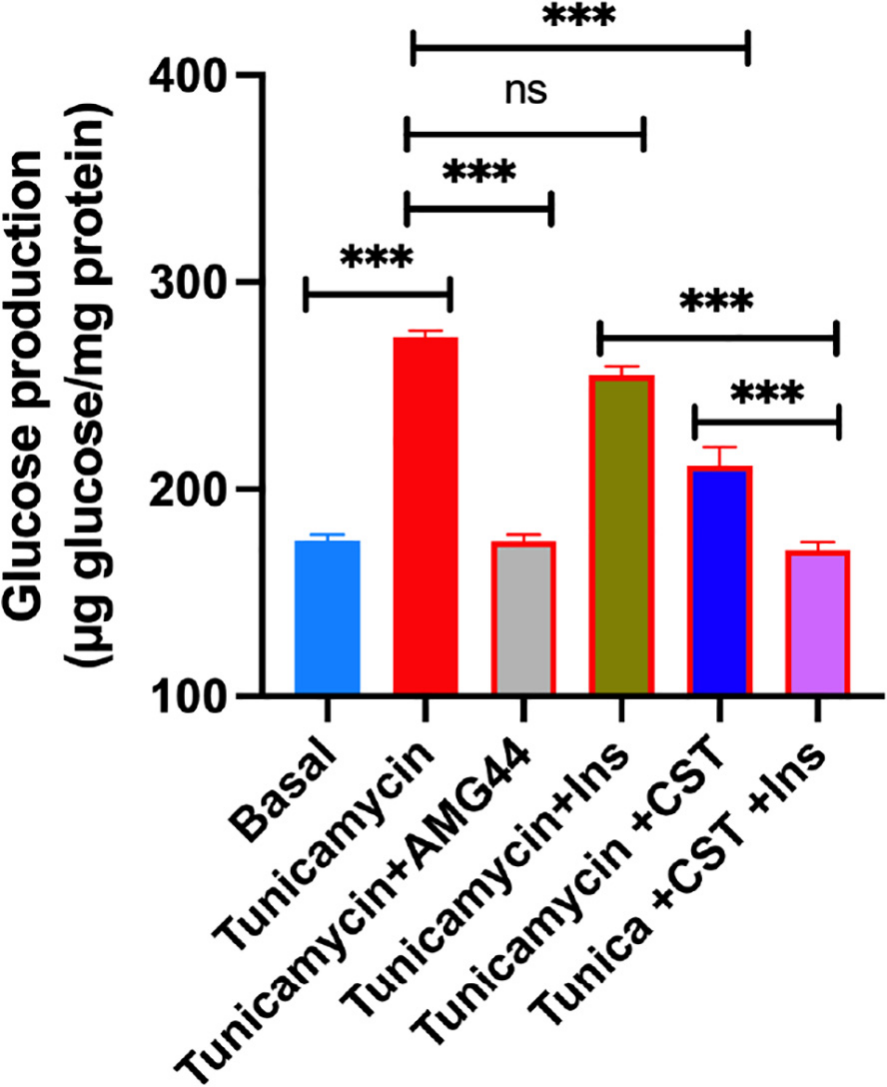
Hepatocyte cultures were exposed to tunicamycin (1 μg/ml) and CST (100 nM, or AMG44 (5 μM) alone or in combination for 12 h. Cultures were then washed twice with glucose-free Krebs–Ringer bicarbonate buffer (HKRB) and incubated with HKRB buffer or insulin (10 nM) in the presence of pyruvate (5 mM) and lactate (10 mM) for another 4 h. At the end of incubation, glucose concentration in the culture medium was measured. Culture plates were washed with PBS and attached cells were extracted with NaOH for protein assay. Glucose values were normalized with protein.

**Fig. 13:**
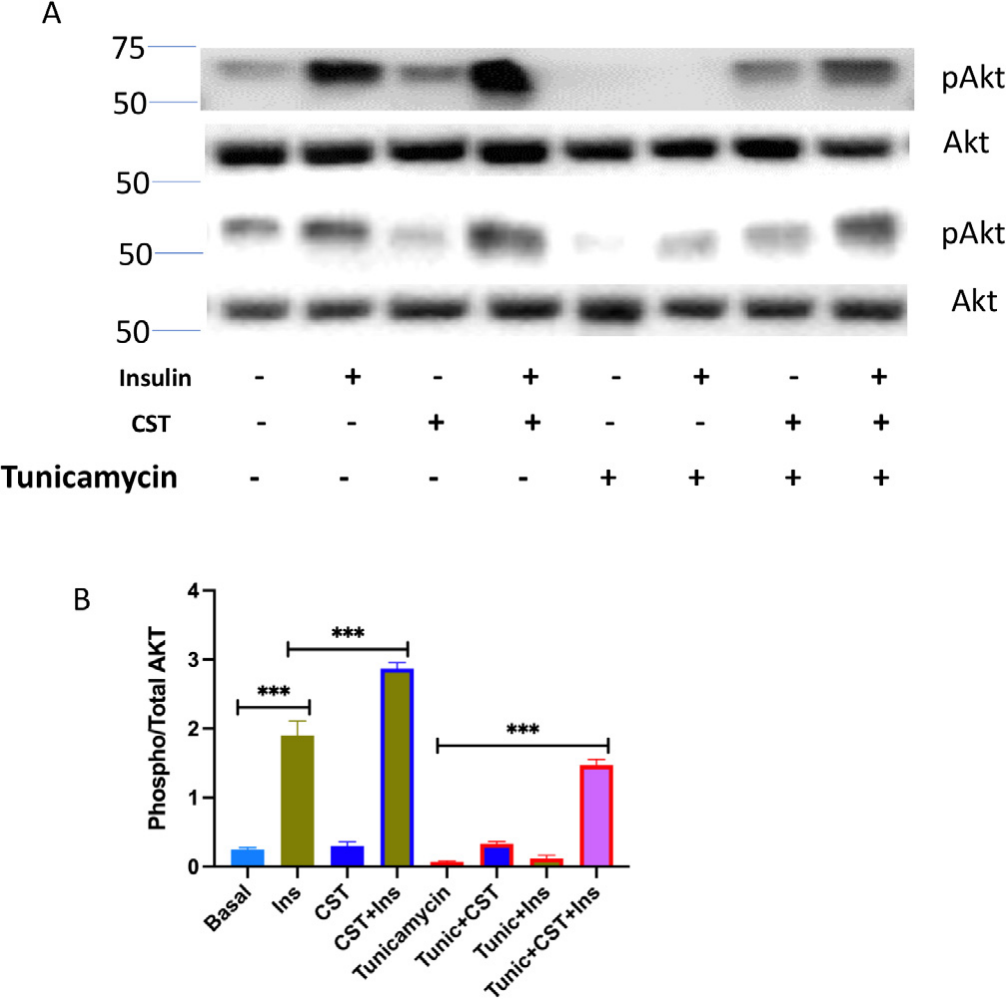
Hepatocytes cultures were treated with saline (Control) and Tunicamycin (Tunic) (1 μg/ml) for 12 h. Then control and tunicamycin treated cultures were treated with CST (100 nM) or AMG44 (5 μM) for 6 h. Then some cultures were treated with insulin (10 nM) for 10 min. At the end cultures were terminated and protein extracts were subjected to western blotting for (A) pAkt(S473) and Akt signals and (B) signal densities were plotted as bar graph. Average of three blots were quantitated.

**Fig. 14:**
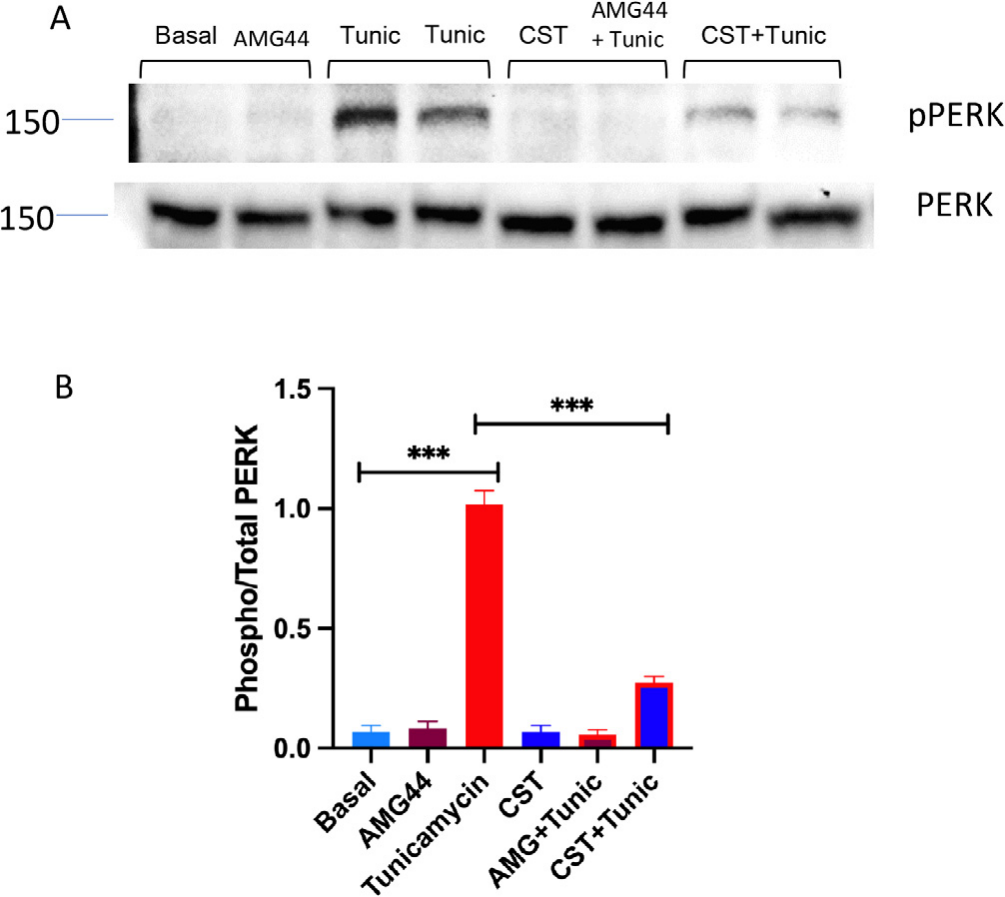
Hepatocytes cultures were treated with saline (Control) and Tunicamycin (Tunic) (1 μg/ml) for 12 h. Then control and tunicamycin treated cultures were treated with CST (100 nM) or AMG44 (5 μM) for 6 h. Then some cultures were treated with insulin (10 nM) for 10 min. At the end cultures were terminated and protein extracts were subjected to western blotting for (A) pPERK and PERK signals, and (B) signal densities were plotted as bar graphs. Average of three blots are quantitated.
(iv) **Low pPERK and low pAKT**
  Here, low signal value for pPERK (Fig. S7E of Supplementary File S1) led to improved insulin sensitivity (Fig. S7G of Supplementary File S1) in spite of low pAKT signal (Fig. S7F of Supplementary File S1). Besides, very low ER stress (Fig. S7G of Supplementary File S1) compared to DIO situation along with low values for the ratios (pPERK/PERK), (pIRE1α/IRE1α) and (peIF2α/eIF2α) (Fig. S7H of Supplementary File S1) were found. Although this condition may arise when CST works in an insulin-independent way, it confirms that pAKT may be a significant marker to be targeted for higher insulin sensitivity by attenuating ER stress because this condition showed lower insulin sensitivity (steady value was near about 1.7 in Fig. S7G of Supplementary File S1) compared to same (steady value near was about 4.7 in Fig. S7C of Supplementary File S1) in “low pPERK and high pAKT” condition mentioned earlier. Experimental verification of such claim can be found in Fig. 12, Fig. 13, Fig. 14 illustrated later.

(i) **High pPERK and low pAKT**
  We found high ER stress and low insulin sensitivity along with high values for the ratios, i.e., (pPERK/PERK), (pIRE1α/IRE1α) and (peIF2α/eIF2α) (Fig. S6C-D of Supplementary File S1) due to a high signal value for pPERK and a low signal value for pAKT (Fig. S6A-B of Supplementary File S1).
(ii) **High pPERK and high pAKT**
  High signal of pAKT (Fig. S6F of Supplementary File S1) was able to enhance insulin sensitivity (Fig. S6G of Supplementary File S1) in spite of high pPERK (Fig. S6E of Supplementary File S1) resulting in high ER stress (Fig. S6G of Supplementary File S1). Besides, the other ratios (pPERK/PERK), (pIRE1α/IRE1α) and (peIF2α/eIF2α) remained high (Fig. S6H of Supplementary File S1). Although, this condition is naturally not possible because of inverse relationship between ER stress (PERK signaling) and Insulin signaling, it clearly reveals that enhancement of pAKT may play a significant role in higher insulin sensitivity overcoming ER stress.
(iii) **Low pPERK and high pAKT**
  Low signal value of pPERK and high signal value of pAKT (Fig. S7A-B of Supplementary File S1), enhanced insulin sensitivity (Fig. S7C of Supplementary File S1). Besides, ER stress became low (Fig. S7C of Supplementary File S1). This condition also depicted low values for the ratios (pPERK/PERK), (pIRE1α/IRE1α) and (peIF2α/eIF2α) (Fig. S7D of Supplementary File S1). The “thought experiments” of previous cases already confirmed that reduction of pPERK resulting in attenuation of ER stress, resembling CST effect, could enhance insulin sensitivity. Subsequently, “high pPERK and high pAKT” condition (although unnatural) discussed earlier gave a clue that enhancement of pAKT may have an effective role in higher insulin sensitivity overcoming ER stress. We will discuss its experimental verification (Fig. 12, Fig. 13, Fig. 14) later.

Finally, it can be concluded that although reduction of pPERK on application of CST drives enhanced insulin sensitivity, pAKT can be treated as another drug target during high ER stress. An experimental condition can be created where the effects of overexpression of active Akt can be tested in an insulin-resistant model. This will be discussed later.

### 3.7 Decreased ER lumen dilation due to CST treatment

Since lipid loading creates a stress condition which might alter ER morphology, we looked at the ER by TEM and found that the ER lumen got dilated in infiltrated DIO liver macrophages (Fig. 11A–C & G), which was reduced markedly by CST (Fig. 11D–G). Whether this could be an independent marker for CST action is yet to be established.

### 3.8 Alleviation of ER stress by CST results in attenuation of inflammation and improvement in insulin signaling

Macrophage-specific deletion of IRE1α conferred resistance to high-fat diet-induced obesity, thereby linking macrophages to ER stress, inflammation and insulin sensitivity [81]. The transcription factor NF-κB is normally suppressed by its inhibitor, IκBα. When IKK (IκB kinase) is activated, it phosphorylates IκBα, which leads to its degradation resulting in activation of NF-κB [82], [83]. Activated NF-κB translocates to the nucleus and augments the expression of proinflammatory genes [84], [85], [86]. ER stress-induced IRE1 physically interacts with IKK, leading to an increase in phosphorylation of IκBα and the concomitant decrease in total levels of IκBα, which results in activation of NF-κB [87]. It appears from the existing literature that during ER stress basal IKK activity, retained by IRE1, and PERK-mediated translation inhibition act in concert to activate NF-κB. Chronic treatment of DIO mice with CST resulted in significant decrease in phosphorylation of NF-κB, indicating attenuation of inflammation (Fig. 11H & I). While tyrosine phosphorylation activates, serine phosphorylation of insulin receptor substrates (IRS) at specific serine residues inhibits insulin signaling. ER stress induces insulin receptor signaling through increasing the serine phosphorylation and decreasing the tyrosine phosphorylation of IRS-1 (IRSpY), leading to insulin resistance [88]. One of the mechanisms by which ER stress could impair insulin action is by the activation of JNK through double-stranded RNA-dependent protein kinase (PKR) [68], via the IRE1/TRAF2/ASK1 [59] or the PERK/ERO1L/CHOP/${\mathrm{IP}}_{3}$R/ASK1 [89] pathways, all of which have been reported to impair insulin receptor signaling [20], [84], [87], [90]. We found significant decrease in phosphorylation of JNK after chronic treatment with CST (Fig. 11H & J), which indicates improvement in insulin signaling. The improvement in insulin signaling by CST is further strengthened by increased phosphorylation of AKT (Fig. 11H & K) and FoxO1 (Fig. 11H & L).

### 3.9 ER stress and insulin sensitivity in hepatocytes are modulated by CST: An alternative model.

Understanding a mechanism in an *in vivo* model is always associated with complications arising from multiple interacting factors. Therefore, interpretation of CST data in *in vivo* situation is expected to have some limitations. In order to get more direct insight, we used primary hepatocyte cultures and induced ER stress using tunicamycin, a known stress inducer. We analysed both functional effect by measuring glucose production as well as signaling effects on Akt and PERK. In this model we tested the direct effects of CST. Hepatocyte glucose production, mimicking hyperglycemia in animals, was induced by the treatment with tunicamycin along with suppression of Akt signaling and enhancement of PERK signaling (Fig. 12, Fig. 13, Fig. 14). AMG44, a known PERK inhibitor [97], blocked tunicamycin-induced PERK phosphorylation (Fig. 14) and tunicamycin-induced glucose production (Fig. 12). Insulin suppressed hepatic glucose production but could not reverse tunicamycin effect suggesting insulin resistance induced by tunicamycin through stimulation of ER stress in the hepatocytes. As expected, tunicamycin inhibited Akt phosphorylation thus explaining why insulin could not suppress tunicamycin-induced glucose production (Figs. 12 & 13). CST partially suppressed tunicamycin effect but in combination with insulin, completely reversed tunicamycin effect on glucose production (Fig. 12). In other words, CST revived the suppressive effect of insulin which insulin could not achieve alone. CST enhanced insulin-induced Akt phosphorylation, and a combination of CST and insulin could restore pAKT signals significantly, which was inhibited completely by tunicamycin (Fig. 13). CST also demonstrated significant inhibition of tunicamycin-induced PERK phosphorylation (Fig. 14). Taken together, the hepatocyte culture model demonstrated the phenotypes of ER stress and its modulation by insulin and CST. It may be noted that the increased Akt-phosphorylation achieved by a combination of insulin and CST (Fig. 13), was able to reverse ER stress mediated (induced by tunicamycin) suppression of insulin sensitivity (suppression of glucose production by insulin (Fig. 12)). This was one of the predictions of *in silico* modelling.

### 3.10 Tyrosine phosphorylation (pY) of insulin receptor (IR) and insulin receptor substrate (IRS) and their relationship with phosphorylation of the ER stress marker PERK

**Fig. 15:**
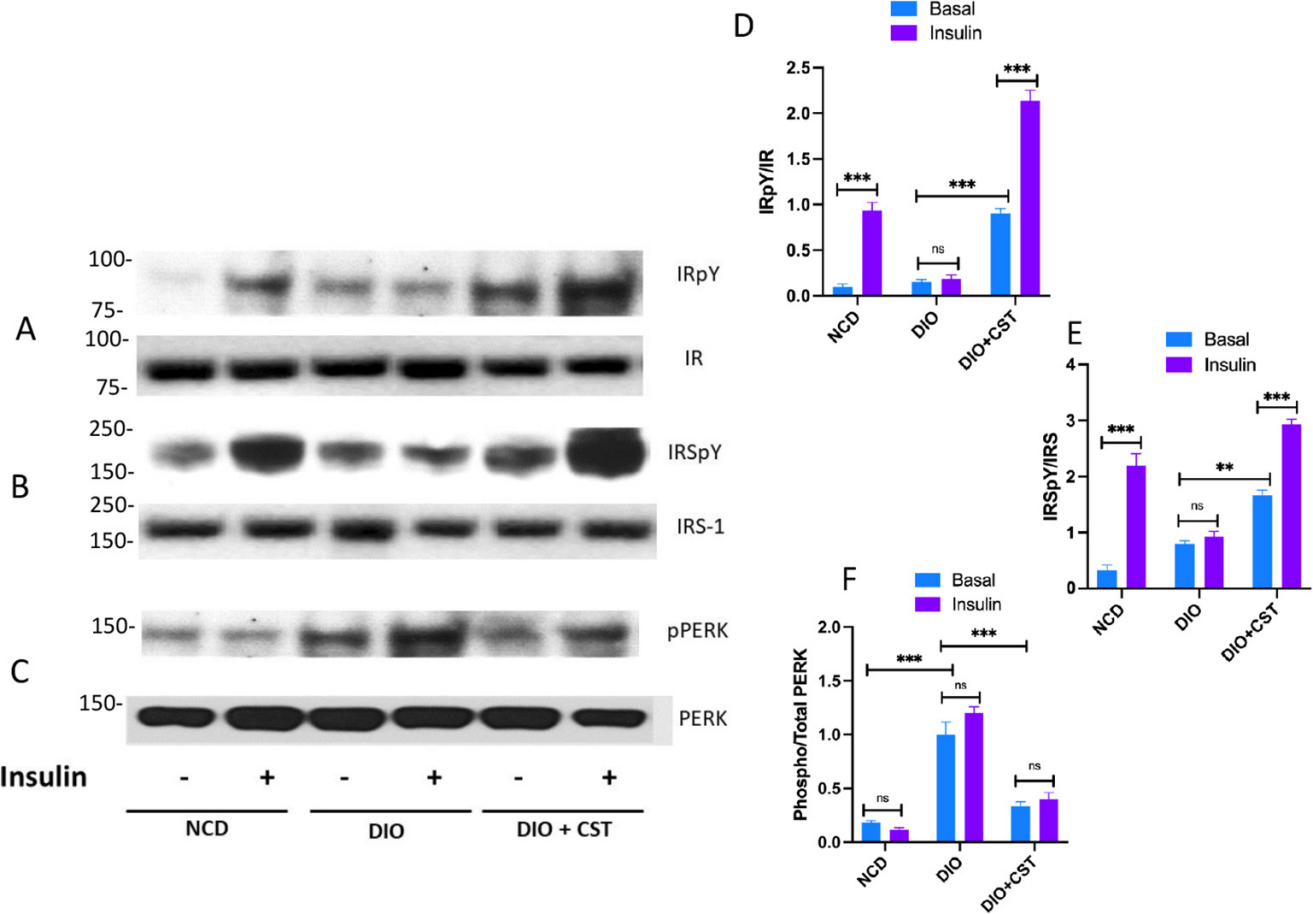
Western blotting of liver samples from, NCD, DIO and CST treated DIO mice. Mice were injected IP with saline or insulin (0.4 mU/g body weight) and sacrificed after 10 min, and tissues were frozen under liquid nitrogen. Liver protein samples were immunoblotted for insulin receptor-phosphotyrosine (IRpY) & insulin receptor (IR) (A & D), insulin receptor substrate-phosphotyrosine (IRSpY) & insulin receptor substrate-1 (IRS-1) (B & E), phospho-PERK and PERK (C & F) signals.

One of the goals of this study with *in silico* modelling of ER stress pathway and insulin signaling pathway is to look at the status of the markers of these pathways so that one can predict a phenotype or a physiological outcome. Another goal is to dissect out a therapeutically important target molecule with the help of CST. We analysed these two pathways both in *ex vivo* hepatocyte culture model as well as in animal model. Using pAkt and pPERK as the markers of insulin signaling pathway and ER stress pathway respectively, we saw an inverse relationship in hepatocytes (Fig. 12, Fig. 13, Fig. 14). Increased stress suppresses insulin-stimulated Akt signaling but if Akt signaling is enhanced by CST treatment, it can prevent stress effect significantly. Similar results were obtained from the animal model of obesity. Obesity induced ER stress, represented by the increased pPERK signals (Fig. 15C & F) suppressed tyrosine phosphorylation of IR and IRS (Fig. 15A & D and B & E), two markers of insulin signaling pathway. Again, CST treatment enhanced tyrosine phosphorylation of IR and IRS concomitant with the suppression of pPERK signals. These results match with some of the predictions from the *in silico* model.

## 4 Discussion

Experimental analyses revealed that chronic treatment of DIO mice with CST results in attenuation of ER stress. Here we hypothesized that ER stress could contribute to the development of insulin resistance utilizing the following pathways: (i) UPR activated transcription factors modulate expression of the gluconeogenic enzymes PEPCK and G6Pase as well as lipogenic enzyme SREBP1c. It has been reported that ER stress increases glucose-6-phosphatase activity and glucose output in primary hepatocytes [91]. This decreased insulin signaling was mediated by activated IRE1, probably through TRAF2 recruitment and JNK activation [59]. Furthermore, gluconeogenesis could be activated either by ER stress-induced inhibition of IL-6/STAT3-dependent suppression of hepatic gluconeogenic enzyme expression [93] or by activated (by ER stress) CREBH-induced augmentation of transcription of gluconeogenic genes [94]. We have reported recently that CST decreased gluconeogenesis by inhibiting expression of PEPCK and G6Pase genes [33] (ii) ER stress induced activation of IRE1 recruits JNK and IKK by recruiting TRAF2 and ASK1 [59], [60], which impair insulin signaling by phosphorylating IRS1 on serine residues. Furthermore, saturated fatty acids, ceramides and ER stress activate PKR, which inhibits insulin signaling by inducing phosphorylation of serine residues in IRS1 (direct regulation) as well as activating JNK signaling pathway (indirect regulation) [68], [95]. Finally, the PERK-induced activation of TRB3, the ER stress-inducible tribbles ortholog in humans also leads to the impairment of insulin signaling by inhibiting AKT (PKB) [96]. Furthermore, NF-κB can also be activated by ER stress-induced PERK pathway and ATF6 branches [92]. (iii) UPR promotes accumulation of fat in hepatocytes by inducing *de novo* lipogenesis (direct effect) and affecting VLDL secretion (indirect effect) resulting in the development of insulin resistance. Here, we found that CST decreased ER lumen diameter in DIO liver, indicating decreased ER stress. Thus, attenuation of ER stress in liver by CST may also contribute to improved insulin sensitivity in addition to inhibition of Ly6C + macrophage infiltration and inflammation as well as suppression of hepatic glucose production [33].

In order to test the above hypothesis, we developed a PID controller based state space model. Firstly, an *in silico* state space model was designed by integrating ER stress and insulin signaling pathways, and validated with the experimental results for both DIO and NCD conditions. The present state space model resembled the experimental behavioral pattern including high ratios of (pPERK/PERK), (pIRE1α/IRE1α) and (peIF2α/eIF2α) during ER stress (DIO). In addition, in consistent with existing literature, the state space model showed decreased insulin sensitivity (represented by pAkt/total Akt signal ratio) along with decreased IRpY, IRSpY, and pFoxO1 signals along with concentration of ${\mathrm{PIP}}_{3}$ due to the activation of pJNK and pIKKβb during ER stress (DIO). Secondly, we applied two PID controllers on the present state space model to explore a significant marker or a combination of significant markers to be targeted for higher insulin sensitivity overcoming ER stress. Simulation results showed that reduction of pPERk, which can be achieved by applying CST on DIO mice, not only alleviated ER stress but also enhanced insulin sensitivity. Experimental findings validated the computationally derived hypothesis. On the other hand, according to *in silico* studies, high level of phosphorylated AKT played a significant role to enhance insulin sensitivity in spite of high ER stress. This prediction generated by our *in silico* model, validated in hepatocyte culture model using different stressor, opens up a scope of succeeding *in vivo* experiment to verify the effectiveness of enhanced AKT phosphorylation to increase insulin sensitivity overcoming high ER stress. However, on application of CST, reduced pPERK, resulting in low ER stress, can increase insulin sensitivity independent of pAKT signal. Thus, we can conclude that CST reduces not only inflammation but also the ER stress and, as a result, increases the insulin sensitivity in obese model. Additionally, we can treat AKT as another drug target to enhance insulin sensitivity during high ER stress.

## Conflict of interest statement

There is no competing interest.
