# Supplementary material for: Catestatin improves insulin sensitivity by attenuating endoplasmic reticulum stress: In vivo and in silico validation
Source: Comput Struct Biotechnol J. 2020 Feb 22;18:464–81. doi: 10.1016/j.csbj.2020.02.005 (PMC7063178; doi:10.1016/j.csbj.2020.02.005)
Supplement: Supplementary file 2 [file mmc2.pdf]

Supplementary File S1

Catestatin improves insulin sensitivity by attenuating endoplasmic reticulum stress: *in vivo* and *in silico* validation

By

Abhijit Dasgupta[5], Gautam K. Bandyopadhyay[3], Indrani Ray[2], Keya Bandyopadhyay[3], Nirmalya Chowdhury[4], Rajat K. De[2*], Sushil K. Mahata[1, 3*]

[1]VA San Diego Healthcare System, 3350 La Jolla Village Drive, CA 92161, USA; [2]Machine Intelligence Unit, Indian Statistical Institute, 203 B.T. Road, Kolkata 700108, India; [3]Department of Medicine, University of California, San Diego, 9500 Gilman Drive, La Jolla, CA 92093-0732, USA; [4]Department of Computer Science and Engineering, Jadavpur University, Kolkata 700032, India; [5]Department of Data Science, School of Interdisciplinary Studies, University of Kalyani, Kalyani, Nadia 741235, West Bengal, India

Short Title: Catestatin regulation of ER stress

Key words: Chromogranin A, catestatin, endoplasmic reticulum stress, insulin sensitivity, obesity, PID controller

***Correspondence should be addressed** either to Rajat K. De, Ph.D., Machine Intelligence Unit, Indian Statistical Institute, 203 B.T. Road, Kolkata 700108, India, Email: rajat@isical.ac.in, or to Sushil K. Mahata, Ph.D., Metabolic Physiology & Ultrastructural Biology Laboratory, Department of Medicine, University of California, San Diego (0732), 9500 Gilman Drive, La Jolla, CA 92093-0732, Tel (858)-552-8585, extension 2637, E-mail: smahata@health.ucsd.edu.

Notes:

1) As discussed in the main article, we have developed an *in silico* state space model based on the integrated ER stress and insulin signaling pathways as depicted in **Figure S1**.

2) Here we have shown the contents of two matlab files.

- "**New_ER_stress_without_CST_ODE_solve.m**" contains the initialization of variables (in red fonts at page 5) and the procedure to solve ordinary differential equations (ODE) under consideration.
- On the other hand, "**ER_stress_Insulin_equations_new.m**" contains all ordinary differential equations for state components and values for kinetic parameters (in red fonts at pages 6-8).

In combination of these two matlab files, the *in silico* state space model has successfully mimicked the experimental behavior related to ER stress as described in the main article.

3) We have applied Proportional-Integral-Derivative (PID) controllers on this state space model ("ERstress_Insulin_pathway" block in **Figure S2**) to investigate the significant markers to be targeted for alleviation of ER stress and enhanced insulin sensitivity. Here we have simulated the model as depicted in **Figure S2** for three cases targeting i) pPERK and IRpY, ii) pPERK and IRSpY and iii) pPERK and pAKT.

4) We have included primer sequences in **Table S1**.

5) **Figure S3**: In silico state space model depicting that signals of intermediate molecules (A) pFoxO1 enhance in control (NCD) in comparison with stress (DIO) condition. On the other hand, the signal value of (B) pNF-κB becomes higher during stress (DIO) than in control (NCD) scenario.

6) **Figure S4**: **High pPERK and low IRSpY.** Here (A) phosphorylated PERK output as well as (B) IRSpY output is controlled by the Proportional-Integral-Derivative (PID) controllers according to the reference input PERK target and IRS target respectively. As a result, (C) shows high ER stress and low insulin sensitivity. Besides, (D) ratios (phosphorylated PERK/total-PERK), (phosphorylated IRE1α/total IRE1α) and (phosphorylated elF2α/total-elF2α) are quite high around 0.8.

High pPERK and high IRSpY. Here (E) phosphorylated PERK output as well as (F) IRSpY output is controlled by Proportional-Integral-Derivative (PID) controllers according to the reference input PERK target and IRS target respectively. As a result, (G) shows high ER stress and low insulin sensitivity. Besides, (H) ratios (phosphorylated PERK/total PERK), (phosphorylated IRE1α/total IRE1α) and (phosphorylated eIF2α/total eIF2α) are quite high around 0.8.

7) **Figure S5**: **Low pPERK and high IRSpY.** Here (A) phosphorylated PERK output as well as (B) IRSpY output is controlled by the Proportional-Integral-Derivative (PID) controller according to the reference input PERK target and IRS target respectively. As a result, (C) shows low ER stress and high insulin sensitivity. Besides, (D) ratios (phosphorylated PERK/total PERK), (phosphorylated IRE1α/total IRE1α) and (phosphorylated eIF2α/total eIF2α) are quite low around 0.5.

Low pPERK and low IRSpY. Here (E) phosphorylated PERK output as well as (F) IRSpY output is controlled by the Proportional-Integral-Derivative (PID) controller according to the reference input PERK target and IRS target respectively. As a result, (G) shows that ER stress is very low, while insulin sensitivity is increased. Besides, (H) ratios (phosphorylated PERK/total PERK), (phosphorylated IRE1α/total IRE1α) and (phosphorylated eIF2α/Total eIF2α) are quite low around 0.5.

8) **Figure S6**: **High pPERK and low pAKT.** Here (A) phosphorylated PERK output as well as (B) phosphorylated AKT output is controlled by the Proportional-Integral-Derivative (PID) controller according to the reference input PERK target and AKT target respectively. As a result, (C) shows high ER stress and low insulin sensitivity. Besides, (D) ratios (phosphorylated PERK/total-PERK), (phosphorylated IRE1α/total IRE1α) and (phosphorylated elF2α/total-elF2α) are quite high around 0.8.

High pPERK and high pAKT. Here (E) phosphorylated PERK output as well as (F) phosphorylated AKT output is controlled by Proportional-Integral-Derivative (PID) controller according to the reference input PERK target and AKT target respectively. As a result, (G) shows high ER stress and insulin sensitivity. Besides, (H) ratios (phosphorylated PERK/total PERK), (phosphorylated IRE1α/total IRE1α) and (phosphorylated eIF2α/total eIF2α) are quite high around 0.8.

9) **Figure S7**: **Low pPERK and high pAKT.** Here (A) phosphorylated PERK output as well as (B) phosphorylated AKT output is controlled by the Proportional-Integral-Derivative (PID) controller according to the reference input PERK target and AKT target respectively. As a result, (C) shows low ER stress and high insulin sensitivity. Besides, (D) ratios (phosphorylated PERK/total PERK), (phosphorylated IRE1α/total IRE1α) and (phosphorylated eIF2α/total eIF2α) are quite low around 0.5.

Low pPERK and low pAKT. Here (E) phosphorylated PERK output as well as (F) phosphorylated AKT output is controlled by the Proportional-Integral-Derivative (PID) controller according to the reference input PERK target and AKT target respectively. As a result, (G) shows that ER stress is very low, while insulin sensitivity is high. Besides, (H) ratios (phosphorylated PERK/total PERK), (phosphorylated IRE1α/total IRE1α) and (phosphorylated eIF2α/Total eIF2α) are quite low around 0.5.

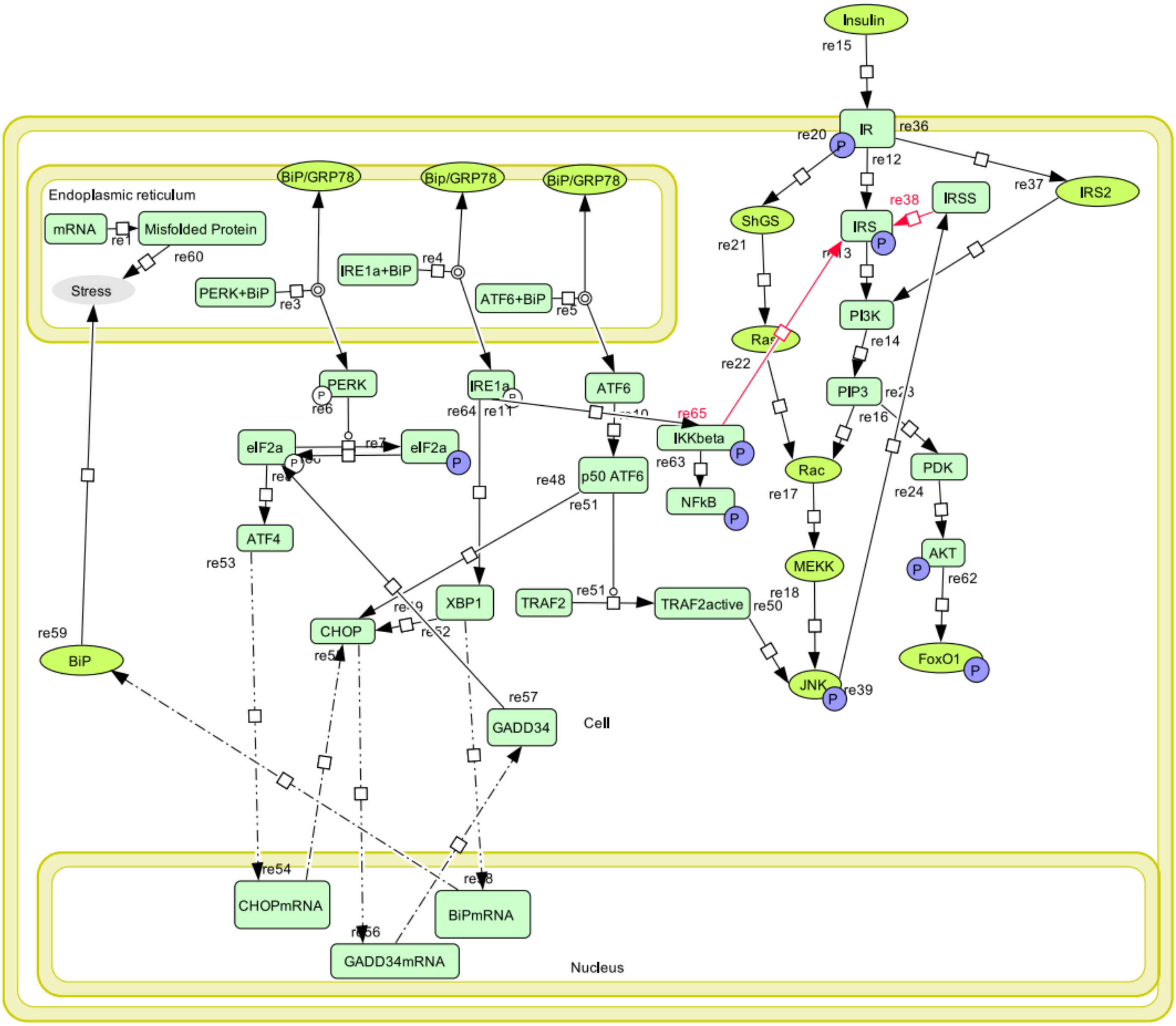

Figure S1. The integrated ER stress and insulin signaling pathways have been considered to develop the *in silico* state space model described in the main article. Here black colored arrow means activation while red colored arrow represents inhibition.

"**New_ER_stress_without_CST_ODE_solve.m**"

```
x_v_Stressed(1:36) = 1.05;    %%% initialization of state components
u_stressed = zeros(1, 2);     %%% initialization of inputs i.e.,stress and insulin
ratio_stressed = zeros(1, 3); %%% initialization of three ratios i.e.,
                              %%% Phosphorylated PERK/Total PERK,
                              %%% Phosphorylated IRE1α/Total IRE1α and
                              %%% Phosphorylated eIF2α/Total eIF2α

%%% Time Span and number of iterations initialization
T  = 10;
Ts = 0.0002;
Tspan = [0 Ts];   %%% Time Span
t = [0:Ts:T]';
i=1;
no_epoch = 70000; %%% Number of iterations

for i = 1:no_epoch
   [xd,ui,ratioi] = ER_stress_Insulin_equations_new(t,x_v_Stressed(i, :));
   u_stressed(i, :)= real(ui(end, :));
   ratio_stressed(i,:)=real(ratioi(end,:));
   [tsim, xsim] = ode23tb('ER_stress_Insulin_equations_new', Tspan,
                              x_v_Stressed(i,:));  %%%% ODE solve
    x_v_Stressed(i+1, :) = real(xsim(end, :));

    %%%%%%%% For restriction the values of state components within a
    %%%%%%%% specific range
    for j=1:36
        if(x_v_Stressed(i+1,j))<1
            x_v_Stressed(i+1,j)=1;
        end
        if(x_v_Stressed(i+1,j))> 5
             x_v_Stressed(i+1,j)= 5;
        end
    end
    fprintf('%d Iteration completed\n\n',i);
end
```

"**ER_stress_Insulin_equations_new.m**"

```
function [xdot,u,ratio] = ER_stress_Insulin_equations_new(t,x_v)
NOM = 36;                % the number of state components
xdot = zeros(NOM, 1);    % rate of changes of state components with respect to time

%%%% molecules initialization
x1    = x_v(1); x2  = x_v(2);  x3   = x_v(3); x4  = x_v(4); x5 = x_v(5);
x6    = x_v(6); x7  = x_v(7);  x8   = x_v(8); x9  = x_v(9); x10= x_v(10);
x11   = x_v(11);x12 = x_v(12); x13  = x_v(13);x14 = x_v(14);
x15   = x_v(15);x16 = x_v(16); x17  = x_v(17);x18 = x_v(18);
x19   = x_v(19);x20 = x_v(20); x21  = x_v(21);x22 = x_v(22);
x23   = x_v(23);x24 = x_v(24); x25  = x_v(25);
x26   = x_v(26);x27 = x_v(27); x28  = x_v(28);
x29   = x_v(29);x30 = x_v(30);
x31   = x_v(31);x32 = x_v(32);
x33 = x_v(33); x34 = x_v(34); x35 = x_v(35); x36 = x_v(36);
```

```
%--------------------------------%
%  Molecule identifier  %
%--------------------------------%

%%%% x1: Misfolded Protein,
%%%% x2:  Bip,
%%%% x3:  PERK+Bip,
%%%% x4:  IRE1α+Bip,
%%%% x5:  ATF6α+Bip,
%%%% x6:  BipmRNA,
%%%% x7:  Phosphorylated PERK,
%%%% x8:  Phosphorylated IRE1α,
%%%% x9:  ATF6α,
%%%% x10: eIF2α,
%%%% x11: Phosphorylated eIF2α,
%%%% x12: ATF4,
%%%% x13: CHOP,
%%%% x14: CHOPmRNA,
%%%% x15: GADD34mRNA,
%%%% x16: GADD34,
%%%% x17: XBP1,
%%%% x18: p50ATF6,
%%%% x19: TRAF2,
%%%% x20: TRAF2active,
%%%% x21: pJNK,
%%%% x22: MEKK,
%%%% x23: Rac,
%%%% x24: Ras,
%%%% x25: ShGS,
%%%% x26: IRpY,
%%%% x27: IRSpY,
%%%% x28: PI3k,
%%%% x29: PIP3,
%%%% x30: IRSS,
%%%% x31: IRS2,
%%%% x32: pIKKβ,
%%%% x33: pNFkB,
%%%% x34: PDK-1,
%%%% x35: Phosphorylated AKT,
%%%% x36: Phosphorylated FoxO1.


%----------------------%
%  Kinetic Parameters  %
%----------------------%

k1  =  2.5;
k2  =  1.011;
k3  =  1.27;
k7  =  1.28;
k8  =  1.21;
k9  =  1.5;
k10 =  1.47;
k11 =  1.50;
k12 =  1.47;
k13 =  1.504;
k14 =  1.51;
k15 =  1.52;
```

```
k16 =  1.201009572 ;
k17 =  1.3034854;
k18 =  1.481008003 ;
k19 =  1.511419;
k64 =  0.021150047513  ;
k21 =  2.878981009157  ;
k22 =  1.05053917922   ;
k65 =  0.0191648;
k23 =  1.3710009595;
k24 =  1.42616557;
k25 =  3.87100357;
k26 =  1.18318491;
k66 =  1.115289 ;
k67 =  1.16416879;
k27 =  1.47100934;
k28 =  1.52416787;
k29 =  1.22007577  ;
k30 =  1.241517431;
k31 =  1.87;
k32 =  1.3519430516555 ;
k33 =  1.296100712 ;
k34 =  1.251000706 ;
k35 =  1.4184319318    ;
k36 =  1.35004 ;
k37 =  1.32951462  ;
k38 =  1.2500971   ;
k39 =  0.115000235 ;
k40 =  1.227126948 ;
k41 =  1.2201003171    ;
k42 =  1.21693199502   ;
k43 =  1.21408100044   ;
k68 =  0.001;
k44 =  0.1720921   ;
k45 =  2.77813 ;
k46 =  0.328201;
k47 =  0.0921  ;
k69 =  2.948934139411078;
k48 =  0.0901009999;
k49 =  2.5441381;
k50 =  0.08101009952456;
k51 =  2.7041421;
k52 =  1.1480310197094 ;
k53 =  1.0063910369151984967547;
k54 =  0.102710761005276;
k55 =  2.62885215154106797 ;
k56 =  2.4164143151    ;
k57 =  0.0925010109    ;
k72 =  0.0911;
k58 =  2.611441741;
k59 =  0.219100991984  ;
k60 =  1.416351634997597   ;
k61 =  1.9010061031013404  ;
k62 =  3.541411065853  ;
k63 =  0.09910105936238;
k_new  = 1.42712;
k_new1 = 1.1001;
k_nfk = 1.523;
k_pdk_1 = 1.897;
k_akt_p = 2.279;
k_Foxo_1_p = 1.9873;
k_pdk_1_decay = 0.4356;
```

```
k_nfk_decay = 0.5987;
k_akt_p_decay = 0.2178;
k_Foxo_1_p_decay = 0.1977;


%%%amplification and feed back constants
F1 = 1.5;
F2 = 1.5;
F3 = 1.05;
F4 = 1.05;
amplify = 1.05;
amplify1= 1;

%%%%% Input equation solve
%u(1) = 0; %%% Zero stress
u(1) = amplify1*((k2*x1*x2)/k1); %%% Non Zero stress
u(2) = amplify*((k52/((1+F3*x30)*(1+F4*x32)))*(x26/k53)); %%% Insulin

%%%%% Specific Ratios
ratio(1)= x7/(x3+x7); %%% Phosphorylated PERK/Total PERK
ratio(2)= x8/(x8+x4);    %%% Phosphorylated IRE1α/Total IRE1α
ratio(3)= x11/(x10+x11); %%% Phosphorylated eIF2α/Total eIF2α



%--------------------%
%  Model Equations   %
%--------------------%

%%%%% ER stress pathway *******
xdot(1)  =  k1*u(1)-k2*x1*x2;
xdot(2)  = -k3*x2 + k7*x6*x1;
xdot(3)  = -k8*x3*x7 + k9*x1*x2;
xdot(4)  = -k12*x4*x8 + k13*x1*x2;
xdot(5)  = -k16*x5*x9 + k17*x1*x2;
xdot(6)  = -k31*x6 + k32*x17*x1;
xdot(7)  =  k10*x3*x1*x2 -k11*x7;
xdot(8)  = -k14*x8 + k15*x4*x1*x2;
xdot(9)  = -k18*x9 + k19*x5*x1*x2;
xdot(10)= -k64*x10*(1+F1*x7)*x1*x2;
xdot(11)= -k21*x11 + k22*x10*(1+F1*x7)*x1*x2 + k65*x16;
xdot(12)= -k23*x12 + k24*x11*x1*x2;
xdot(13)= -k25*x13 + k26*x14*x1*x2 + k66*x17*x1*x2+ k67*x18*x1*x2;
xdot(14)= -k27*x14 + k28*x12*x1*x2;
xdot(15)= -k29*x15 + k30*x13*x1*x2;
xdot(16)=  k33*x15*x1*x2 -k34*x16;
xdot(17)=  k35*x8*x1*x2 -k36*x17;
xdot(18)=  k37*x9*x1*x2 -k38*x18;
xdot(19)= -k39*x19*(1+F2*x18)*x1*x2;
xdot(20)=  k40*x19*(1+F2*x18)*x1*x2 - k41*x20;
xdot(21)=  k42*x20*x8*x1*x2 + k68*x22 - k43*x21;
xdot(32)=  k_new*x8*x1*x2 - k_new1*x32;
xdot(33)  = k_nfk*x32*x1*x2 - k_nfk_decay*x33;


%%%%%%%%%%%%%%%%%%%%%%%%%%%%%%%%%%%%%%%%%%%%%%%%%%%%%%%%%%%%%%%
%%%%% Insulin signaling pathway *******
xdot(22)= -k44*x22 + k45*x23/((1+F3*x30)*(1+F4*x32));
xdot(23)= -k46*x23 + k47*x24/((1+F3*x30)*(1+F4*x32)) +
```

```
            k69*x29/((1+F3*x30)*(1+F4*x32));
xdot(24)= -k48*x24 + k49*x25/((1+F3*x30)*(1+F4*x32));
xdot(25)= -k50*x25 + (k51*x26)/((1+F3*x30)*(1+F4*x32));
xdot(26)= -k52*x26/((1+F3*x30)*(1+F4*x32)) + (k53* u(2));
xdot(27)= (-k54*x27)*((1+F3*x30)*(1+F4*x32)) + (k55*x26);
xdot(28)= (k56*x27)/((1+F3*x30)*(1+F4*x32))+ k72*x31/((1+F3*x30)*(1+F4*x32)) -
          k57*x28;
xdot(29)= k58*x28/((1+F3*x30)*(1+F4*x32)) - k59*x29;
xdot(30)= k60*x21*x8*x1*x2 - k61*x30;
xdot(31)= (k62*x26)/((1+F3*x30)*(1+F4*x32)) - k63*x31;
xdot(34) = k_pdk_1*x29/((1+F3*x30)*(1+F4*x32))-k_pdk_1_decay*x34;
xdot(35) = k_akt_p*x34/((1+F3*x30)*(1+F4*x32))-k_akt_p_decay*x35;
xdot(36) = k_Foxo_1_p*x35/((1+F3*x30)*(1+F4*x32))-k_Foxo_1_p_decay*x36;



end
```

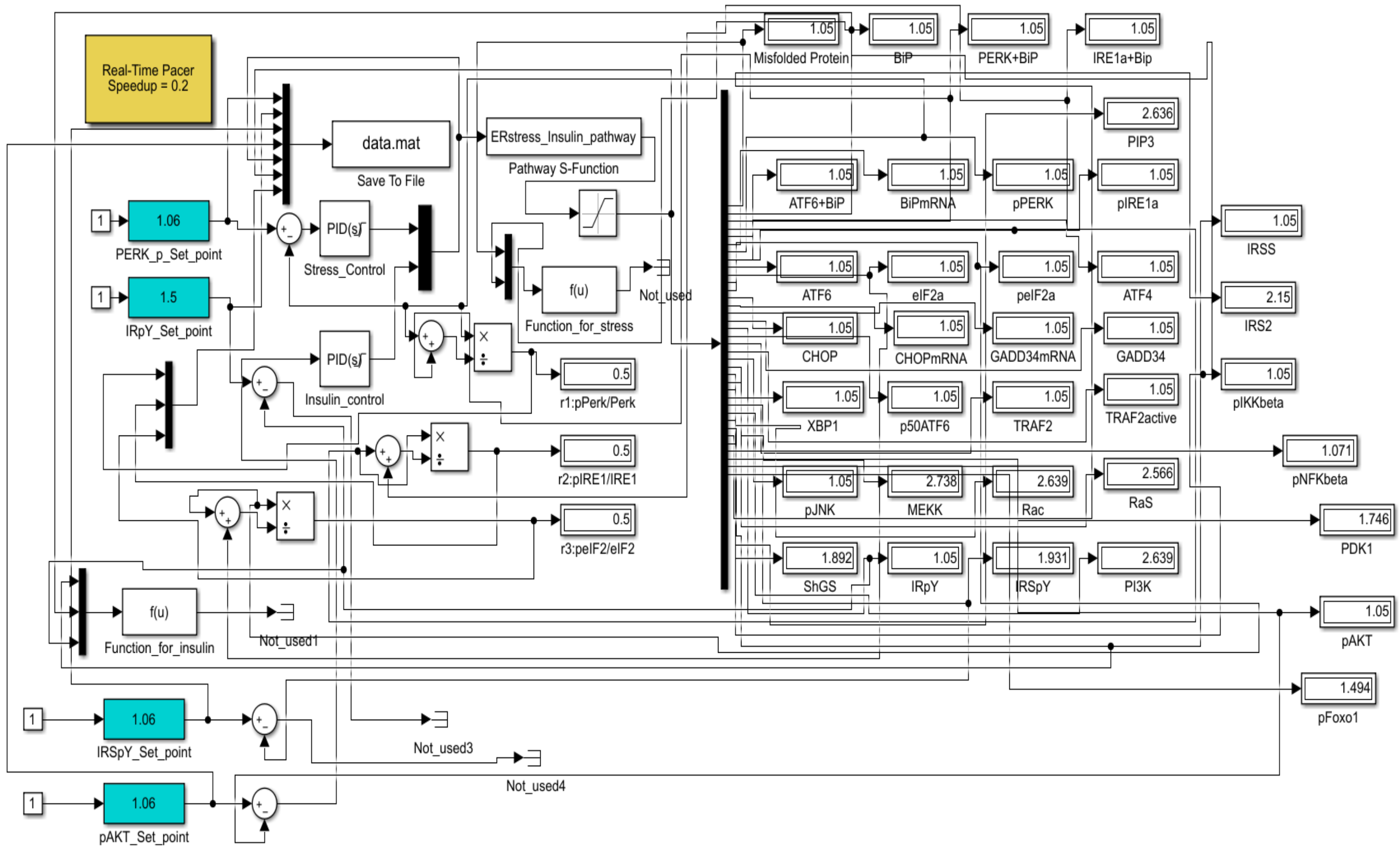

Figure S2. Proportional-Integral-Derivative (PID) controllers have been applied on the state space model "ERstress_Insulin_pathway" to control the outputs of IRpY, IRSpY, pAKT and pPERK to certain desired low/high values. According to the error, appropriately calculated control (external) inputs have been applied for ER stress and insulin.

Table S1. Primer sequences for genes used in the real-time PCR analysis

Description	Sequence 5'-3'	GeneBank Accession
Atf4-FP	CCTGAACAGCGAAGTGTTGG	NM_009716
Atf4-RP	TGGAGAACCCATGAGGTTTCAA	
Atf6a-FP	TCGCCTTTTAGTCCGGTTCTT	NM_001081304
Atf6a-RP	GGCTCCATAGGTCTGACTCC	
Gapdh-FP	AGGTCGGTGTGAACGGATTTG	NM_008084
Gapdh-RP	TGTAGACCATGTAGTTGAGGTCA	
Xbp1-us-FP	AGCTTTTACGGGAGAAAACTCAC	NM_013842
Xbp1-us-RP	CCTCTGGAACCTCGTCAGGA	
Xbp1-s-FP	GAGTCCGCAGCAGGTG	NM_013842
Xbp1-s-RP	GTGTCAGAGTCCATGGGA	

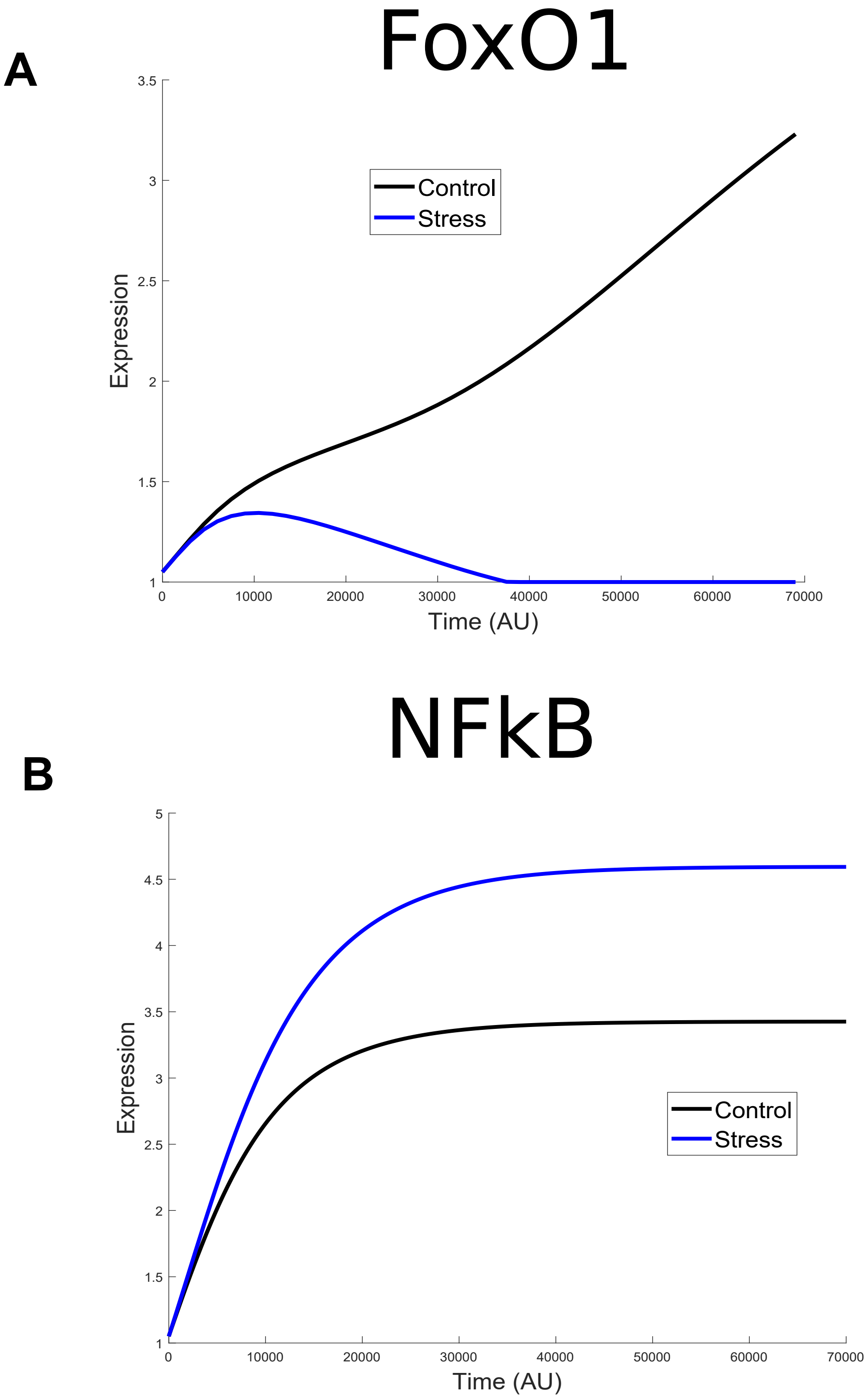
A
FoxO1
Control
Stress
Expression
Time (AU)
B
NFkB
Control
Stress
Expression
Time (AU)

Figure S3.

High PERK and low IRST

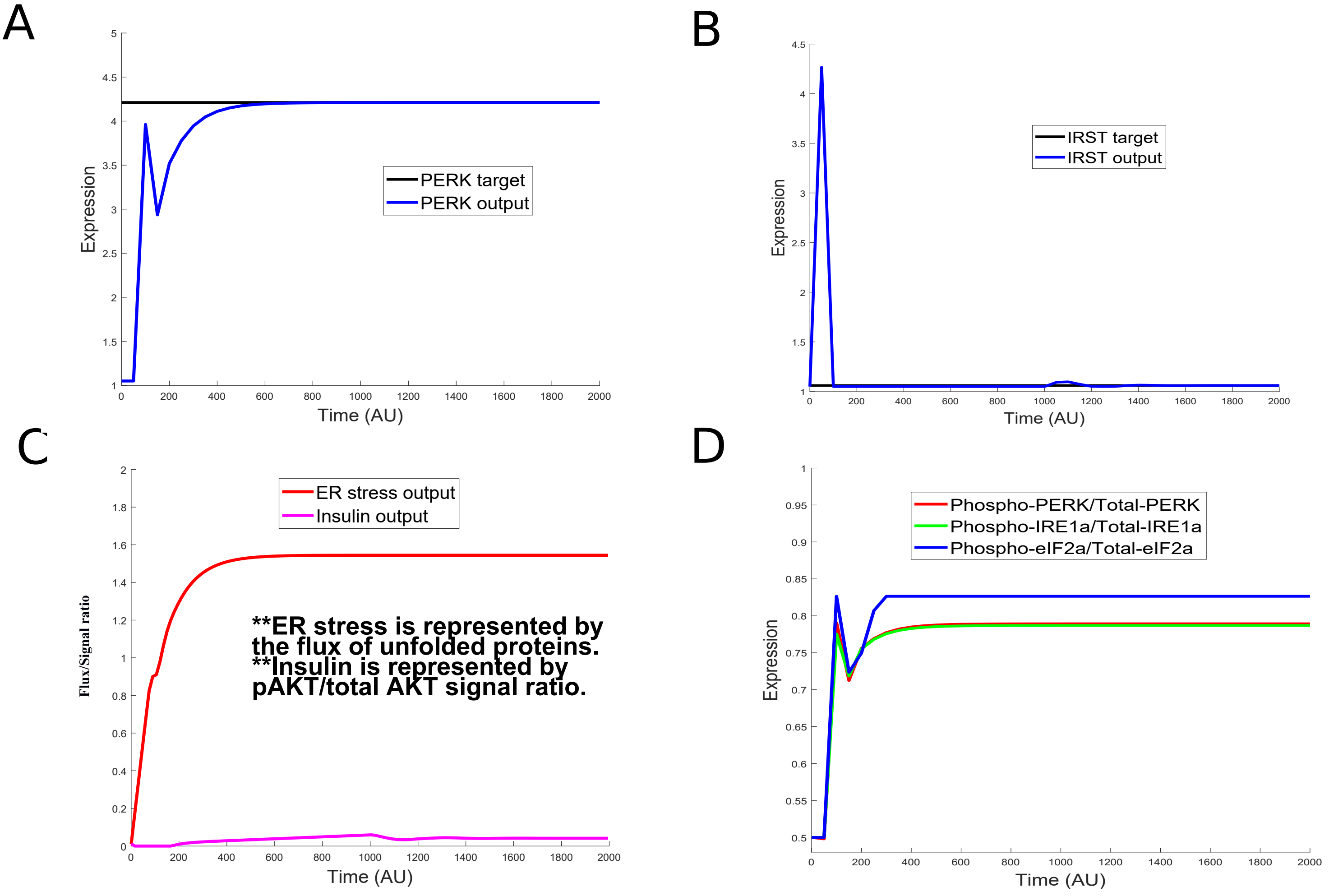

High PERK and high IRST

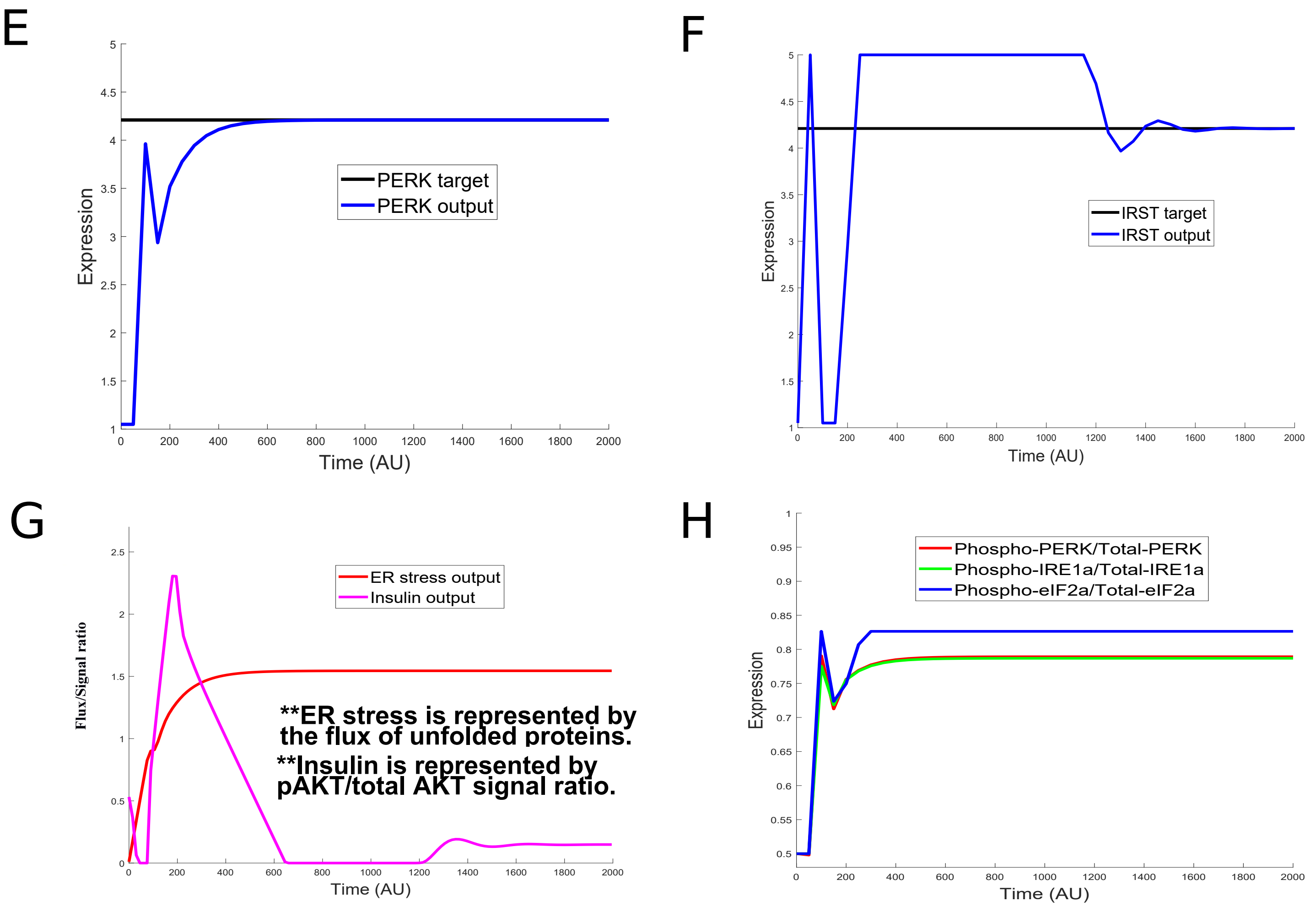

Figure S4.

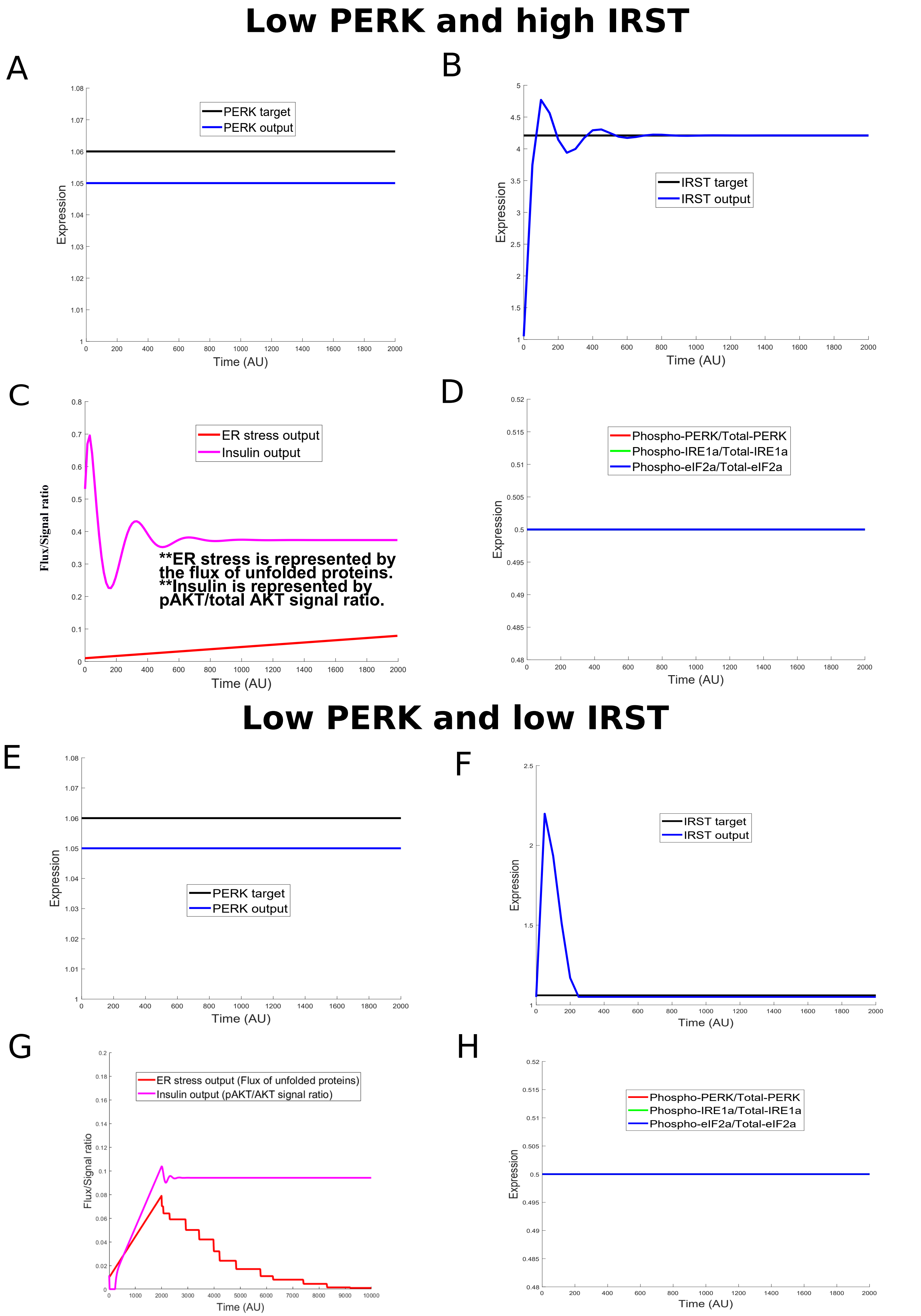

Low PERK and high IRST
A
PERK target
PERK output
Expression
Time (AU)
B
IRST target
IRST output
Expression
Time (AU)
C
ER stress output
Insulin output
**ER stress is represented by the flux of unfolded proteins.
**Insulin is represented by pAKT/total AKT signal ratio.
Flux/Signal ratio
Time (AU)
D
Phospho-PERK/Total-PERK
Phospho-IRE1a/Total-IRE1a
Phospho-eIF2a/Total-eIF2a
Expression
Time (AU)
Low PERK and low IRST
E
PERK target
PERK output
Expression
Time (AU)
F
IRST target
IRST output
Expression
Time (AU)
G
ER stress output (Flux of unfolded proteins)
Insulin output (pAKT/AKT signal ratio)
Flux/Signal ratio
Time (AU)
H
Phospho-PERK/Total-PERK
Phospho-IRE1a/Total-IRE1a
Phospho-eIF2a/Total-eIF2a
Expression
Time (AU)

Figure S5.

High PERK and low AKT

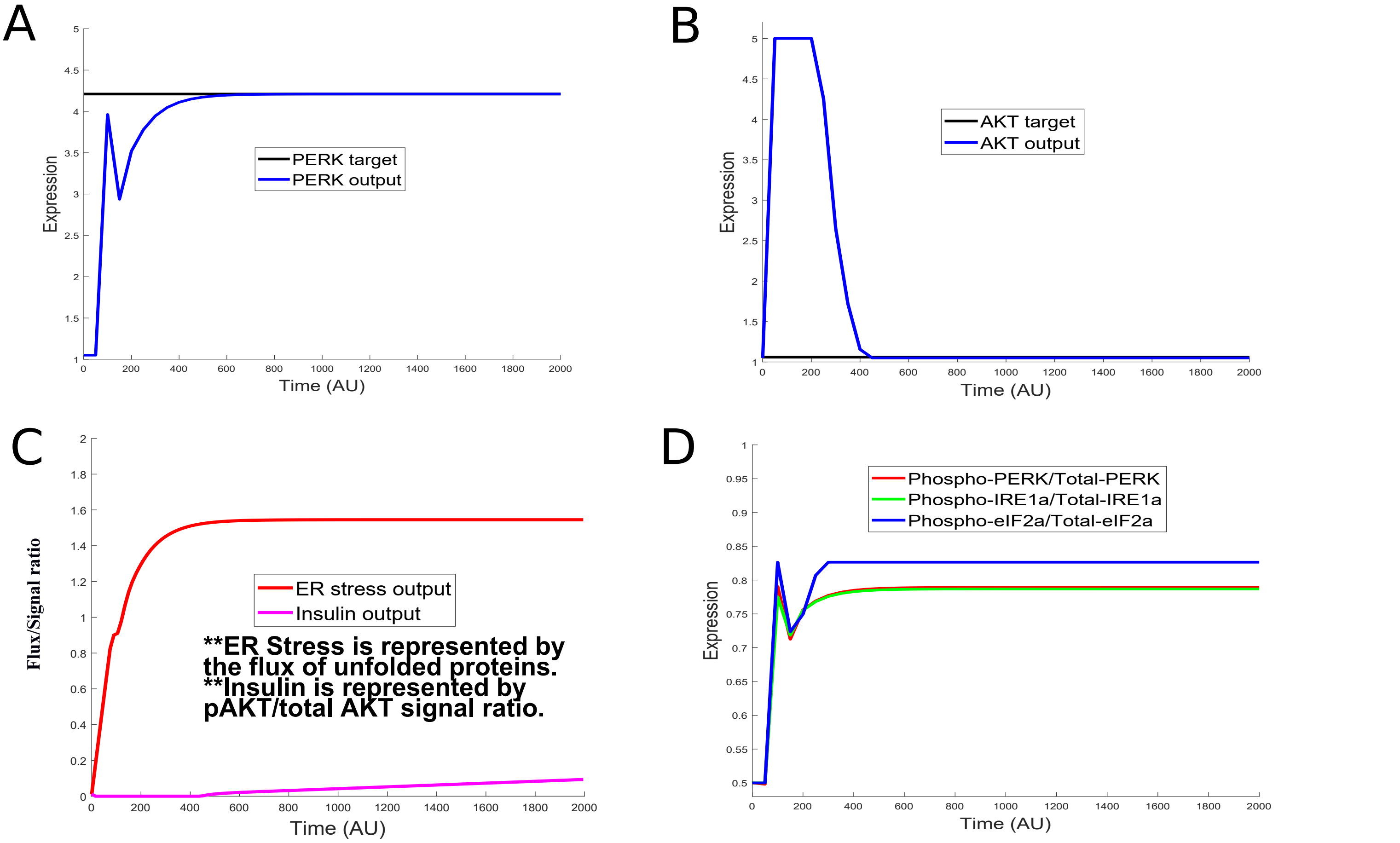

High PERK and high AKT

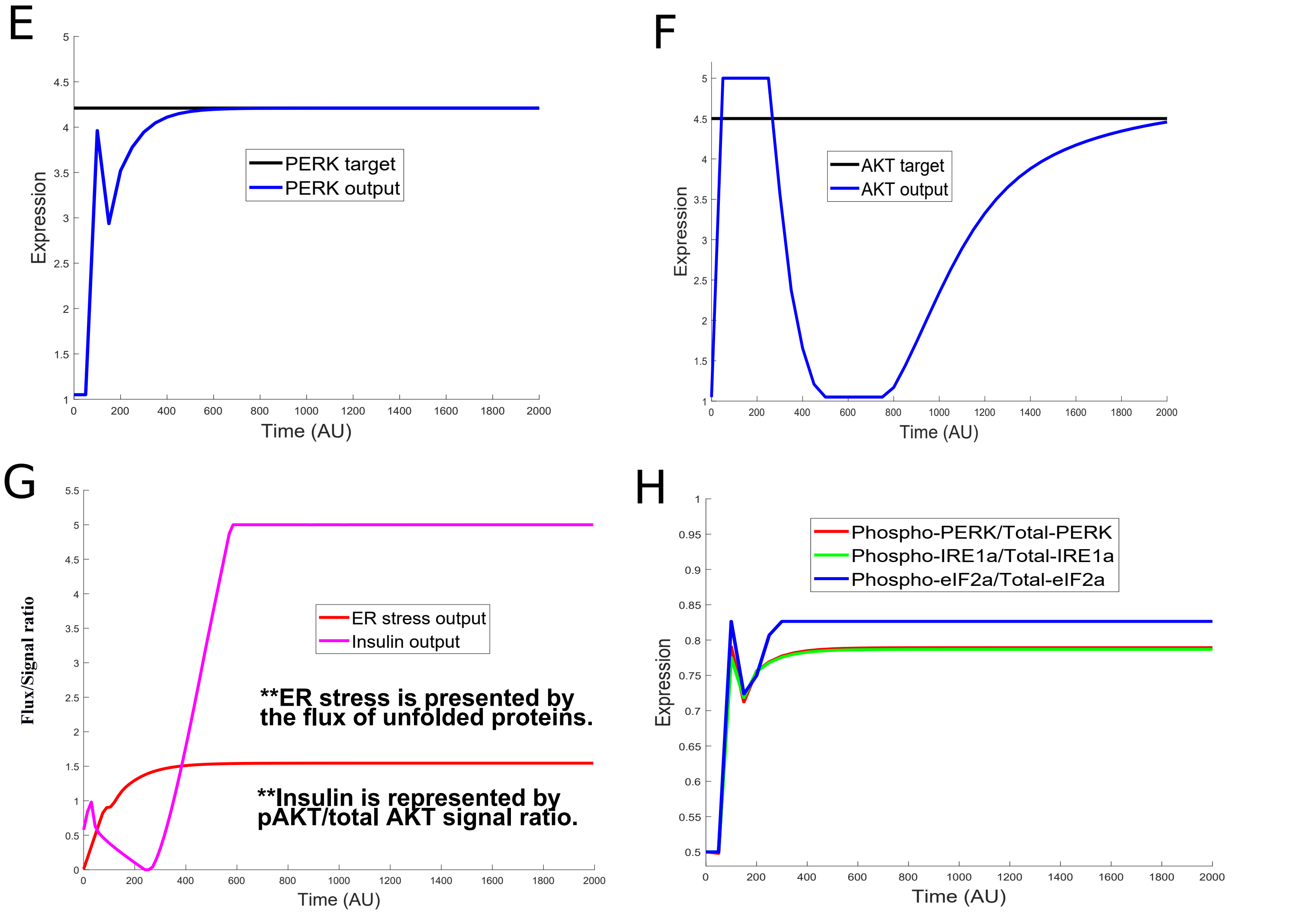

Figure S6.

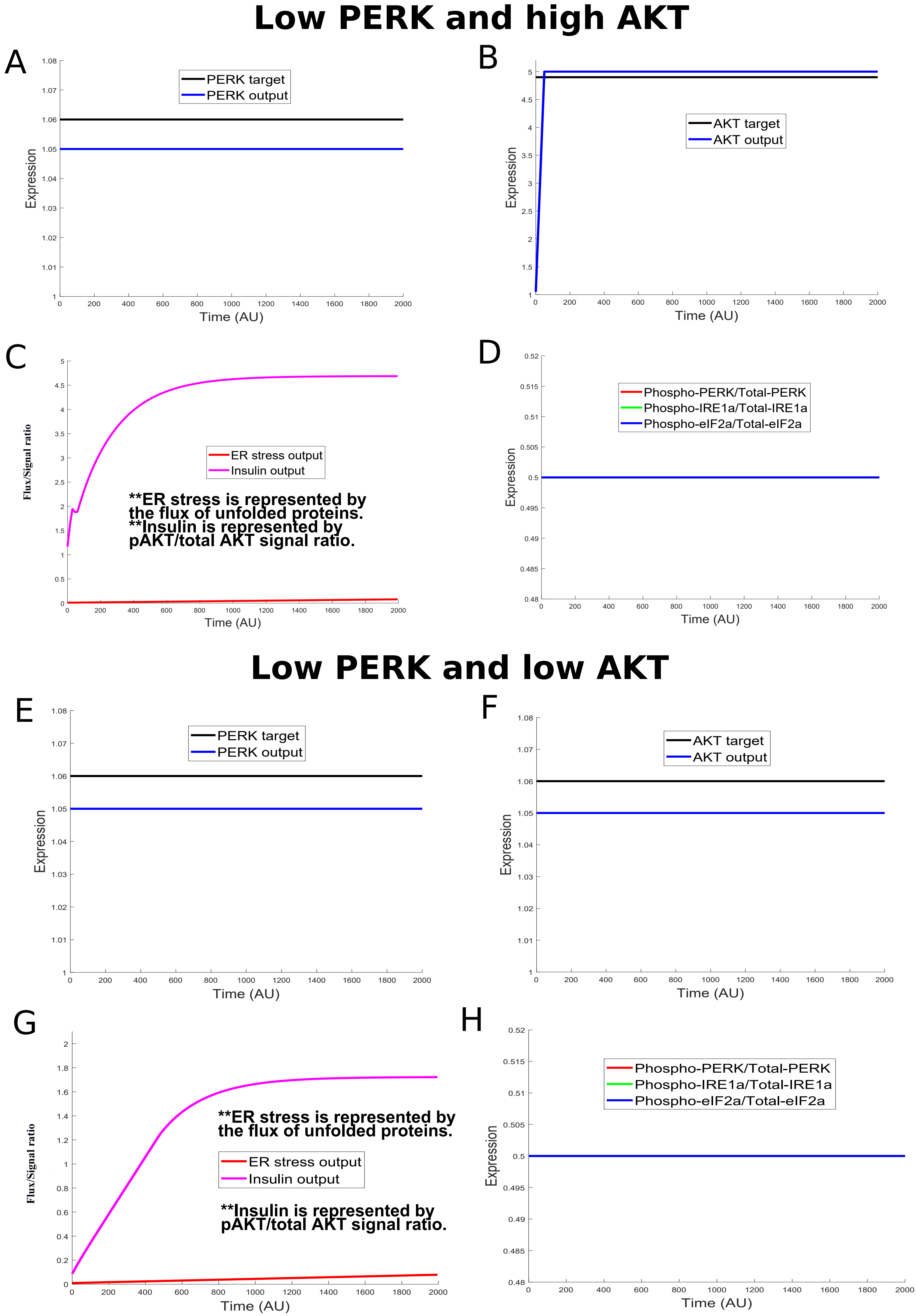

Low PERK and high AKT
A
PERK target
PERK output
Expression
Time (AU)
B
AKT target
AKT output
Expression
Time (AU)
C
ER stress output
Insulin output
**ER stress is represented by the flux of unfolded proteins.
**Insulin is represented by pAKT/total AKT signal ratio.
Flux/Signal ratio
Time (AU)
D
Phospho-PERK/Total-PERK
Phospho-IRE1a/Total-IRE1a
Phospho-eIF2a/Total-eIF2a
Expression
Time (AU)
Low PERK and low AKT
E
PERK target
PERK output
Expression
Time (AU)
F
AKT target
AKT output
Expression
Time (AU)
G
**ER stress is represented by the flux of unfolded proteins.
ER stress output
Insulin output
**Insulin is represented by pAKT/total AKT signal ratio.
Flux/Signal ratio
Time (AU)
H
Phospho-PERK/Total-PERK
Phospho-IRE1a/Total-IRE1a
Phospho-eIF2a/Total-eIF2a
Expression
Time (AU)

Figure S7.
